# Heuristic exploitation of genetic structure in marker-assisted gene pyramiding problems

**DOI:** 10.1186/s12863-014-0154-z

**Published:** 2015-01-30

**Authors:** Herman De Beukelaer, Geert De Meyer, Veerle Fack

**Affiliations:** Department of Applied Mathematics, Computer Science and Statistics, Ghent University, Krijgslaan 281 - S9, Gent, 9000 Belgium; Bayer CropScience NV, Innovation Center, Technologiepark 38, Zwijnaarde, 9052 Belgium

**Keywords:** Plant breeding, Marker-assisted gene pyramiding, Multi-objective optimization, Heuristics

## Abstract

**Background:**

Over the last decade genetic marker-based plant breeding strategies have gained increasing attention because genotyping technologies are no longer limiting. Now the challenge is to optimally use genetic markers in practical breeding schemes. For simple traits such as some disease resistances it is possible to target a fixed multi-locus allele configuration at a small number of causal or linked loci. Efficiently obtaining this genetic ideotype from a given set of parental genotypes is known as the marker-assisted gene pyramiding problem. Previous methods either imposed strong restrictions or used black box integer programming solutions, while this paper explores the power of an explicit heuristic approach that exploits the underlying genetic structure to prune the search space.

**Results:**

Gene Stacker is introduced as a novel approach to marker-assisted gene pyramiding, combining an explicit directed acyclic graph model with a pruned generation algorithm inspired by a simple exhaustive search. Both exact and heuristic pruning criteria are applied to reduce the number of generated schedules. It is shown that this approach can effectively be used to obtain good solutions for stacking problems of varying complexity. For more complex problems, the heuristics allow to obtain valuable approximations. For smaller problems, fewer heuristics can be applied, resulting in an interesting quality-runtime tradeoff. Gene Stacker is competitive with previous methods and often finds better and/or additional solutions within reasonable time, because of the powerful heuristics.

**Conclusions:**

The proposed approach was confirmed to be feasible in combination with heuristics to cope with realistic, complex stacking problems. The inherent flexibility of this approach allows to easily address important breeding constraints so that the obtained schedules can be widely used in practice without major modifications. In addition, the ideas applied for Gene Stacker can be incorporated in and extended for a plant breeding context that e.g. also addresses complex quantitative traits or conservation of genetic background. Gene Stacker is freely available as open source software at http://genestacker.ugent.be. The website also provides documentation and examples of how to use Gene Stacker.

**Electronic supplementary material:**

The online version of this article (doi:10.1186/s12863-014-0154-z) contains supplementary material, which is available to authorized users.

## Background

Over the last decade several genetic marker-based plant breeding strategies [1] have been established and are increasingly used to develop better lines and hybrids. The approach taken depends on trait architecture. For simple traits such as some disease or pest resistances it is possible to tag a small number of causal or linked loci with genetic markers and exploit these by marker-assisted selection [2]. More complex traits such as yield are better managed by thousands of genome-wide markers focusing on prediction [3] rather than on causality of individual markers. At present, genotyping technologies are no longer limiting and the major challenge is to optimally use genetic markers in practical breeding schemes.

Exploitation of genetic markers through crossing and selection is a combinatorial optimization problem in a genetic context. This problem has two distinct levels of objectives. For foreground markers that address simple traits, a fixed multi-locus allele configuration or genetic ideotype is targeted. Complex trait objectives managed by background markers require a more general optimization in a constrained space. At the same time, there is the need to deal with crop specific as well as practical constraints such as the expected amount of seeds obtained from a crossing, the number of generations, and the number of plants grown per generation. In all, the number of objectives and their diverse types make this a hard and complex problem that demands an explicit and modular optimization strategy.

**Figure 1:**
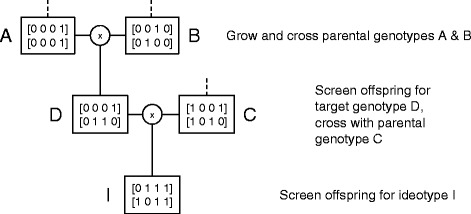
**General crossing schedule layout (example).** In each generation, a number of plants are grown and screened for the desired target genotype(s). Crossings are then performed to provide new offspring to be grown, genotyped and selected for use in the next generation. All targets and crossings are fixed in advance. This example has 3 parental genotypes **A**, **B** and **C**. Initially, **A** and **B** are crossed to produce an intermediate target genotype **D**. In the next generation, **D** is crossed with parental genotype **C** to create the ideotype **I**.

A logical first step is to develop an explicit framework to deal with the foreground markers. The objective is to design a crossing schedule that efficiently stacks a small number of favorable trait alleles (causal or tightly linked) present in a set of parental genotypes. This is known as the marker-assisted gene pyramiding or gene stacking problem. A crossing schedule consists of a number of generations in which plants are grown and screened to identify desired genotypes or targets. These targets are selected for crossings, generating offspring to be grown, genotyped and selected for use in the next generation, until the ideotype is obtained. An example with 3 parental genotypes is given in Figure 1. The number of possible crossing schedules grows exponentially with the number of loci and parental genotypes which makes the task of designing good schedules very challenging. With *n* loci, every single crossing may produce a vast amount of up to $\mathcal {O}(4^{n})$ possible offspring which are all candidates to be fixed as target genotypes in the next generation. There are two main aspects that define a crossing schedule: the target genotypes aimed for in each generation (selection problem) and the crossings to be performed with these selected targets (scheduling problem). Important properties of a crossing schedule are the number of generations (time) and the number of plants (cost) required to obtain the target genotypes in each generation. The latter is inversely proportional to the probability of observing these targets among the offspring.

Previous research on this topic has mainly focused on providing general guidelines for plant breeders [4,5] while only few papers offer a systematic algorithmic approach. An initial method [6] considered restricted parental genotypes and represented crossing schedules as binary trees. For each crossing, the progeny that inherits all favorable alleles from both parents is selected, i.e. the selection problem is not addressed. An exhaustive algorithm is applied to generate all possible crossing schedules by iteratively combining smaller schedules through additional crossings. Later, integer programming approaches were developed that optimize using general purpose solvers like CPLEX^a^. The first implementation [7] performs a multi-objective optimization to fix desirable alleles while maintaining genetic variability at some remaining loci when possible. Only the selection problem is considered: each target allele in the ideotype is assigned an originating parental genotype and arbitrary minimum-depth binary trees are used to stack the genes according to this assignment. In a more powerful mixed integer programming (MIP) implementation [8] crossing schedules are modeled as directed acyclic graphs (DAGs) that allow reuse of material. Both the selection and scheduling problem are considered and the model incorporates a constraint on the number of offspring generated from one crossing.

**Our contribution:** We introduce Gene Stacker as a novel approach to marker-assisted gene pyramiding, combining an explicit DAG model [8] with a pruned generation algorithm inspired by a simple exhaustive search [6]. We demonstrate that this works for small problems while more complex problems require supplementary heuristic pruning criteria that exploit the genetic structure to skip additional, well-chosen parts of the search space. The proposed heuristics provide an interesting quality-runtime tradeoff. This makes Gene stacker not only a flexible and performant tool for many practical problems but also a core module that can be extended to optimize for e.g. quantitative traits.

## Methods

First, several definitions and formulas are stated and an extended DAG model is introduced. Then, the applied optimization strategy is described for which some exact and heuristic pruning criteria are proposed.

### Encoding of genotypes

A diploid phase-known genotype *G*=(*G*_1_,…,*G*_*k*_) consists of an ordered sequence of *k*≥1 chromosomes, each represented by a 2×*n*_*i*_ matrix *G*_*i*_ of alleles. The rows *G*_*i*,1_ and *G*_*i*,2_ of matrix *G*_*i*_ are called haplotypes and each correspond to one of both homologous chromosomes. Note that interchanging the haplotypes (rows) of a chromosome *G*_*i*_∈*G* yields the same genotype. The columns *G*_*i*_(*j*),*j*=1,…,*n*_*i*_, correspond to the considered loci in chromosome *G*_*i*_ and binary digits (0/1) indicate the absence or presence of specific alleles. At every locus 0≤*j*≤*n*_*i*_−1 of chromosome *G*_*i*_ there are two alleles *G*_*i*,1_(*j*) and *G*_*i*,2_(*j*); this *j*th locus is homozygous if *G*_*i*,1_(*j*)=*G*_*i*,2_(*j*), else it is heterozygous. A genotype is said to be homozygous^b^ if all considered loci in each chromosome are homozygous.

### Recombination rates

When crossing two diploid genotypes *P* and *Q*, each parent produces a haploid gamete and fusion of these gametes yields the diploid genotype of the child. A gamete *H*=(*H*_1_,…,*H*_*k*_) produced by genotype *P*=(*P*_1_,…,*P*_*k*_) consists of a series of haploid chromosomes *H*_*i*_ which each comprise a single haplotype and which are each (independently) obtained from the respective diploid chromosome *P*_*i*_. A diploid chromosome can yield a number of different haplotypes due to recombination of alleles (crossover events). The probability with which each possible haplotype is produced can be computed using the genetic map, from which the distance between any pair of loci on the same chromosome can be inferred. These distances are converted to crossover rates *r*_*i*,*p*,*q*_ corresponding to the probability that a crossover will occur between loci *p* and *q* on chromosome *i*, e.g. using Haldane’s mapping function [9]. Then, the probability *Pr*[*P*_*i*_,*Q*_*i*_→*G*_*i*_] that chromosomes *P*_*i*_ and *Q*_*i*_ will yield haplotypes which together form chromosome *G*_*i*_ is computed using formulas introduced in [8] (Additional file 1: Section 1). As Gene Stacker explicitly models multiple chromosomes, the final probability *Pr*[*P*,*Q*→*G*] of producing the entire phase-known genotype *G* when crossing parents *P* and *Q* is computed by multiplying the chromosome probabilities:

$$Pr[P,Q \rightarrow G] = \prod_{i=1}^{k} Pr[P_{i}, Q_{i} \rightarrow G_{i}]. $$

### Population size

Each genotype among the possible outcome of a crossing is a candidate to be selected in the next generation. However, such target genotype can only be selected if it actually occurs among the offspring. Thus, a sufficient amount of offspring should be generated so that the targets are expected to be produced. Consider a crossing of genotypes *P* and *Q* and a target genotype *G* that is produced with probability *ρ*=*Pr*[*P*,*Q*→*G*]. Given a desired success rate *γ*^′^, the corresponding population size *N*(*ρ*,*γ*^′^) indicates the number of offspring that has to be generated so that the probability of obtaining at least one occurrence of *G* is at least *γ*^′^ [6,8]:

(1) $$ N(\rho, \gamma')=\left\{ \begin{aligned} &\left\lceil\frac{\log{(1-\gamma')}}{\log{(1-\rho)}}\right\rceil\quad \text{if}\ \rho < 1 \\ &1 \qquad\qquad\qquad\quad \text{otherwise} \end{aligned}\right.   $$

Gene Stacker ensures a global success rate *γ* (e.g. 95%) by setting a success rate $\gamma ' = \sqrt [n]{\gamma }$ for each individual target, where *n* is the total number of targets obtained from crossings that can produce more than one possible child (i.e. crossings with uncertainty about the outcome). The total population size of a crossing schedule is equal to the sum of the population sizes required to obtain all target genotypes aimed for through the schedule and reflects the cost of the schedule. When several different genotypes or multiple occurrences of a specific genotype are targeted among offspring grown from a shared seed lot, it is possible to compute a (lower) joint population size expressing the number of offspring that has to be generated to simultaneously obtain all targets (Additional file 1: Section 2).

### Extended DAG model

Gene Stacker models crossing schedules as directed acyclic graphs (DAGs) with three types of nodes:

- *Seed lot nodes*: represent seeds obtained from a crossing, modelling the probability distribution of all phase-known genotypes that may be produced. The source nodes of the graph are seed lot nodes from which the parental genotypes are grown. These initial seed lot nodes are assumed to be genetically uniform, i.e. they contain only one fixed phase-known genotype, and never to be depleted. Every internal seed lot node has a single crossing node as its parent. Edges leaving from a seed lot node are directed towards one or more plant nodes in any subsequent generation.
- *Plant nodes*: represent target genotypes aimed for among offspring grown from a specific seed lot. A plant node is labeled with its phase-known genotype and required population size (groups of plant nodes that are simultaneously obtained from the same seed lot are labeled with the required joint population size instead). If more than one occurrence of the respective genotype is targeted, the desired number of duplicates is indicated. Every plant node has a single seed lot node as its parent. Edges leaving from plant nodes lead to crossing nodes in the same generation.
- *Crossing nodes*: represent crossings with plants from the same generation, resulting in a seed lot available as from the next generation. A crossing node is labeled with the number of times that the crossing will be performed (if more than once). Every crossing node has two (not necessarily distinct) plant nodes as its parents. A single edge leaves from every crossing node to a seed lot node in the next generation.

**Figure 2:**
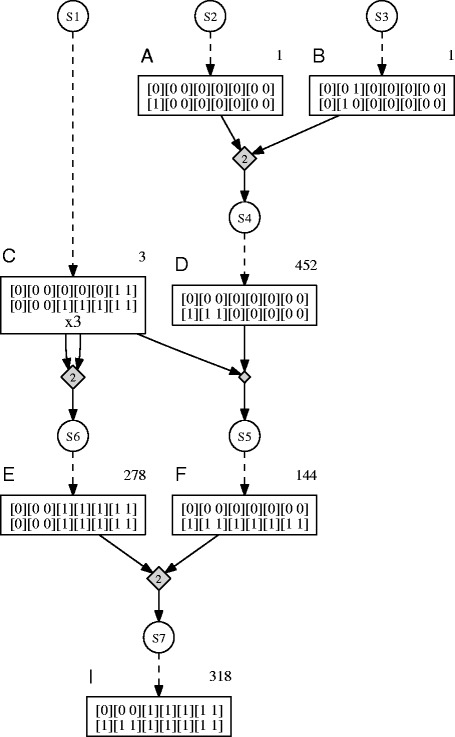
**Example crossing schedule.** An example crossing schedule according to Gene Stacker’s DAG model, with 3 generations and a total population size of 1197 (sum of population sizes required to obtain all target genotypes, as indicated at the corresponding plant nodes). It is assumed that every crossing yields about 250 seeds and that each plant can be crossed twice (or selfed once). First, parental genotypes **A** and **B** are crossed. This crossing is performed twice to provide a sufficient amount of seeds to obtain the target genotype **D** among the offspring. Subsequently, **D** is crossed with the third parental genotype **C** and the latter is also crossed with itself (twice). To be able to perform each of these crossings, 3 duplicates of **C** are grown. Finally, **E** and **F** are crossed (twice) to produce the ideotype **I**.

Figure 2 shows a crossing schedule with 3 generations and a total population size of 1197. It is assumed here that every crossing provides about 250 seeds and that each plant can be crossed twice. Circular nodes represent seed lot nodes, rectangular nodes are plant nodes and diamonds are crossings. Nodes which are aligned at the same vertical level are part of the same generation. The source nodes cover the 0th generation, and each subsequent level of seed lot nodes starts the next generation. This model allows reuse of plants (within a generation) as well as remaining seeds (across generations) and is an extension of the original DAG model from [8] which uses a single node type corresponding to Gene Stacker’s plant nodes.

### Linkage phase ambiguity

Gene Stacker is entirely based on phase-known genotypes as this allows to infer the distribution of possible offspring from a crossing. However, in practice, the linkage phase of a genotype can not always be directly observed [10]. Therefore, it is important to monitor the linkage phase ambiguity (LPA) which expresses the probability that a genotype will have an undesired linkage phase. The observed allelic frequencies of a genotype *G* are referred to as $\widetilde {G}$. When crossing genotypes *P* and *Q*, the probability $Pr[P,Q \rightarrow \widetilde {G}]$ of obtaining any genotype with the same allelic frequencies as *G* is computed as follows:

$$Pr[P,Q \rightarrow \widetilde{G}] = \sum_{G', \widetilde{G}' = \widetilde{G}} Pr[P,Q \rightarrow G']. $$

Then, the linkage phase ambiguity of *G* is equal to

$$LPA[P,Q \rightarrow G] = 1 - \frac{Pr[P,Q \rightarrow G]}{Pr[P,Q \rightarrow \widetilde{G}]}. $$

For target genotypes with non-zero linkage phase ambiguity, the inferred LPA is included in the label of the corresponding plant node. The overall LPA of a crossing schedule is defined as the probability that at least one target genotype aimed for through the schedule will have an undesired linkage phase, and can easily be computed from the individual ambiguities.

### Approximated Pareto frontier

Gene Stacker approximates the *Pareto frontier* of crossing schedules with minimum number of generations, total population size and overall linkage phase ambiguity, possibly subject to a number of crop specific and practical constraints. Upper limits can be set for

- the number of generations (required);
- the overall linkage phase ambiguity;
- the total number of crossings;
- the population size per generation; and
- the number of crossings with each plant.

Also, the expected number of seeds obtained from a crossing can be specified. The Pareto frontier contains all solutions within the constraints that are not dominated by any other valid solution, where *C*^′^ dominates *C* if it is at least as good for every objective and better for at least one objective. All non-dominated schedules are optimal in some sense as they provide tradeoffs with respect to the different objectives. The approximated Pareto frontier obtained by Gene Stacker contains all completed schedules for which no dominating other solution has been constructed.

### Algorithm

Gene Stacker applies a (heuristically) pruned generation algorithm inspired by the exhaustive search strategy from [6]. The search space is traversed as a tree by starting with the smallest possible schedules, i.e. those which simply grow one of the parental genotypes, and iteratively extending schedules through additional crossings. There are two types of extensions: (a) selfing the final plant of a schedule (i.e. crossing this plant with itself); or (b) combining two schedules through a crossing of the final plants of both schedules. Every phase-known genotype among the offspring is then considered to be fixed as the next target, which results in a (possibly large) number of extended schedules.

When combining two schedules, their generations can be aligned or interleaved in different ways (Additional file 1: Section 3). Plant or seed lot nodes occurring in both schedules which are aligned in the same generation of the combined schedule are dynamically reused. Gene Stacker greedily discards non Pareto optimal alignments; therefore, the main algorithm is not exact. However, the impact of this greedy approach on the solution quality is expected to be very small; it mainly prevents the introduction of most likely redundant generations and favors alignments with the highest amount of reuse which leads to a reduced cost.

If an extension yields a new schedule in which the ideotype is obtained, the Pareto frontier is updated accordingly. Else, the schedule is queued for further extension unless it is predicted that every completed extension will either be dominated by an already obtained solution or violate the constraints. Such pruning reduces the number of constructed schedules and therefore the runtime and memory footprint of the algorithm. Gene Stacker includes a number of heuristics that further reduce the search space by exploiting the underlying genetic structure to skip non promising branches of the search tree. Well-designed heuristics may result in large speedups with only a slightly higher probability of obtaining suboptimal solutions, which are often close to the optimum.

The search terminates when there are no more schedules to be further extended. Termination is guaranteed because of a required constraint on the number of generations. A more detailed description of the algorithm is provided in the Additional file 1 (Section 4).

### Exact pruning criteria

Because the number of generations, total population size and overall linkage phase ambiguity are monotonically increasing, any partial schedule which is dominated by a previously obtained solution or which already violates the corresponding constraints may be discarded. In addition, some basic bounds are applied; for example, when combining two partial schedules, it is predicted whether this may yield a valid improvement over the current Pareto frontier approximation by inferring the minimum combined population size and linkage phase ambiguity from the set of non overlapping plant nodes and seed lot nodes occurring in both schedules. Also, the minimum increase in population size and ambiguity caused by targeting any genotype among the offspring of the performed crossing is taken into account. Although these are local bounds that predict the impact of a single extension, they often cause significant speedups as creating all extensions is a time consuming process.

Constructed seed lots are filtered based on the constraints. Genotypes with higher linkage phase ambiguity than the maximum allowed overall ambiguity are removed. Also, if at most *m* plants per generation are allowed, a genotype *G* obtained from crossing *P* and *Q* is discarded if

$$Pr[P,Q \rightarrow G] < 1 - (1-\gamma')^{\frac{1}{m}}. $$

Given that at most *g* generations are allowed, Gene Stacker prunes a significant number of branches when creating schedules with *g*−1 or *g* generations. At generation *g*−1 only genotypes from which the ideotype can be obtained through a single crossing are considered as possible targets, i.e. genotypes that can produce one of both desired haplotypes for every chromosome of the ideotype. Furthermore, in this penultimate generation, only those crossings which can produce the complete ideotype are performed. Obviously, in the final generation *g*, only the ideotype itself is considered as a target. These pruning criteria are very effective and yield huge speedups in the final levels of the search tree.

### Heuristics

Some heuristics are proposed that exploit the underlying genetic structure to further reduce the search space. Several of these heuristics are based on improvement of phase-known genotypes as compared to the ideotype. Improvement is expressed within a chromosome and a genotype is considered to be an improvement if at least one chromosome has improved. Gene Stacker uses two different improvement criteria: *weak* and *strong* improvement. First, the definitions of desired alleles and stretches are introduced.

#### Definition 1 (desired allele).

Given a chromosome *C* with *k* loci, take any of both haplotypes *H* of *C*; then allele *H*(*l*), 0≤*l*≤*k*−1, is desired if the respective chromosome *T* from the ideotype contains a haplotype *H*^′^ with the same allele at locus *l*, i.e. *H*(*l*)=*H*^′^(*l*).

#### Definition 2 (desired stretch).

Given a chromosome *C* with *k* loci, take any of both haplotypes *H* of *C*; then the stretch $S^{H}_{i,j}$, with 0≤*i*,*j*≤*k*−1, *i*≤*j*, is defined as the part of *H* comprising the consecutive alleles at loci *i*,*i*+1,…,*j*. The length of the stretch is denoted as $|S^{H}_{i,j}| = j-i+1$. Stretch $S^{H}_{i,j}$ is desired if the respective chromosome *T* from the ideotype contains a haplotype *H*^′^ for which ∀*l*,*i*≤*l*≤*j*,*H*(*l*)=*H*^′^(*l*).

The definition of weak improvement then follows:

#### Definition 3 (weak improvement).

Given two chromosomes *C*,*C*^′^ and the ideotype , *C* is a weak improvement over *C*^′^, denoted as $C \succ ^{\mathcal {I}}_{w} C'$, if either (a) one of both haplotypes *H* of *C* contains a desired stretch $S^{H}_{i,j}$ which is not present in any of both haplotypes of *C*^′^; or (b) *C* homozygously contains a desired allele which does not occur in *C*^′^ in homozygous state.

The first case favors the introduction of new or extended desired stretches and the second case rewards *stabilization* of desired alleles to prevent them from being lost during subsequent crossings. An alternative definition, of strong improvement, is stated below:

#### Definition 4 (strong improvement).

Given any chromosome *C*, compute the set *M* containing all desired stretches $S^{H}_{i,j}$ occurring in any haplotype *H* that can be produced from *C* with at most 1 crossover. Then derive the tuple (*l*_*C*_,*p*_*C*_) defined by

$$l_{C} = \max\{|S^{H}_{i,j}|; S^{H}_{i,j} \in M\} $$

and

$$p_{C} = \max\{Pr[C \rightarrow S^{H}_{i,j}]; S^{H}_{i,j} \in M~\&~|S^{H}_{i,j}| = l_{C}\} $$

where $Pr[C \rightarrow S^{H}_{i,j}]$ is the probability that *C* will produce any haplotype containing stretch $S^{H}_{i,j}$. Now, given two chromosomes *C*,*C*^′^ and the ideotype ; then *C* is a strong improvement over *C*^′^, denoted as $C \succ ^{\mathcal {I}}_{s} C'$, if

$$(l_{C} > l_{C'})~\vee~(l_{C} = l_{C'}~\wedge~p_{C} > p_{C'}). $$

To detect strong improvement chromosomes are first compared based on the length of the longest desired stretch that may be produced with at most 1 crossover, an idea which has been previously proposed in [11]. In case of equal lengths, the highest probability with which any such maximal desired stretch will be produced by each chromosome is compared. Gene Stacker includes three heuristics which are based on improvement of genotypes. The first heuristic (H0) is applied once to filter the parental genotypes .

#### Heuristic H0 (parental genotype filter).

Discard any parental genotype $G \in \mathcal {G}$ for which $\exists G' \in \mathcal {G}, G' \ne G,$ with $G' \succ ^{\mathcal {I}}_{w} G~\wedge ~\neg (G \succ ^{\mathcal {I}}_{w} G')$.

The other heuristics are repeatedly applied to prune non promising branches of the search tree.

#### Heuristic H1 (improvement over ancestors).

Each genotype *G* is required to be an improvement over all ancestors, i.e. $G \succ ^{\mathcal {I}}_{\ldots } A$ for each genotype *A* occurring on any path from a source node to *G*. It is also allowed that *G*=*A* if *G* has a smaller linkage phase ambiguity or higher probability than *A*, considering the seed lots from which both genotypes are obtained. The applied improvement criterion $\succ ^{\mathcal {I}}_{\ldots }$ can be either weak (H1a) or strong improvement (H1b).

#### Heuristic H2 (seed lot filter).

When crossing genotypes *P* and *Q*, discard any genotype *G* from the obtained seed lot  for which $\exists G' \in \mathcal {S},~G' \ne G, $ with

$$G' \succ^{\mathcal{I}}_{\ldots} G~\wedge~\neg(G \succ^{\mathcal{I}}_{\ldots} G') $$

and both

$$\begin{array}{@{}rcl@{}} Pr[P,Q \rightarrow G'] &\ge& Pr[P,Q \rightarrow G]\\ LPA[P,Q \rightarrow G'] &\le& LPA[P,Q \rightarrow G]. \end{array} $$

Again, the applied improvement criterion $\succ ^{\mathcal {I}}_{\ldots }$ can be either weak (H2a) or strong improvement (H2b).

Heuristic H2 removes genotypes from  if a strictly better genotype is also available which requires equal or less effort to be obtained from , in terms of population size (probability) and linkage phase ambiguity. The following heuristic (H3) assumes that an optimal schedule consists of optimal subschedules.

#### Heuristic H3 (optimal subschedules).

A distinct Pareto frontier $\mathcal {F}(G)$ is maintained for each genotype *G*, consisting of schedules with final genotype *G*. Such schedule *C* is only queued for further extension if it is not dominated by a previous schedule $C' \in \mathcal {F}(G)$. Moreover, extensions are only constructed if *C* is still contained in $\mathcal {F}(G)$ when it is dequeued. As an exception, selfing a homozygous genotype is always allowed.

The exception allows efficient reuse of homozygous genotypes across generations with only a small increase in the number of explored branches of the search tree. Experiments showed that applying heuristic H3 generally results in very large speedups, but regularly also yields worse Pareto frontier approximations because the assumption that optimal schedules consist of optimal subschedules does not hold when reusing material. Therefore, two dual run strategies have been designed where H3 is enabled in the first run only. The second run then benefits from the availability of an initial Pareto frontier approximation, e.g. allowing earlier pruning. Heuristic H3s1 follows this basic dual run strategy. Heuristic H3s2 also applies an additional seed lot filter in the second run that restricts the possible haplotypes for each chromosome to those occurring in a solution found in the first run. The overhead of the first run is usually much smaller than the speedup obtained in the second run.

The next heuristic (H4) requires that a genotype is obtained from a Pareto optimal seed lot in terms of the corresponding probability and ambiguity.

#### Heuristic H4 (Pareto optimal seed lot).

Each genotype *G* is required to be obtained from a Pareto optimal seed lot  in terms of probability and linkage phase ambiguity, among all seed lots available up to the respective generation.

The number of possible offspring from a crossing grows exponentially with the number of (heterozygous) loci in the parents; therefore, it can take a significant amount of time and memory to construct the entire seed lot. Although Gene Stacker includes several seed lot filters, this filtering may also be time consuming. Therefore, heuristics are provided that reduce the number of haplotypes produced from the crossed genotypes’ chromosomes by not considering all crossovers. These heuristics (H5 and H5c) assume that a crossover is difficult to obtain and should therefore result in an obvious improvement.

#### Heuristic H5 (heuristic seed lot construction).

Take a chromosome *C* with *k* loci of which *l*≤*k* are heterozygous with ordered indices *s*=(*ν*_1_,…,*ν*_*l*_). Also, take a haplotype *H* that is produced from *C* through *m*<*l* crossovers between consecutive heterozygous loci $(\nu _{i_{1}-1}, \nu _{i_{1}}), \dots, (\nu _{i_{m}-1}, \nu _{i_{m}})$. Split *H* into a series of *m*+1 corresponding stretches

$$\mathcal{H} = (S^{H}_{0,(\nu_{i_{1}})-1}, S^{H}_{\nu_{i_{1}},(\nu_{i_{2}})-1}, \ldots, S^{H}_{\nu_{i_{m}},k-1}) $$

where each stretch $S^{H}_{i,j} \in \mathcal {H}$ originates from one of both haplotypes of *C*. For every stretch $S^{H}_{i,j}$ originating from the top haplotype *C*_1_, i.e. $S^{H}_{i,j} = S^{C_{1}}_{i,j}$, the bottom haplotype *C*_2_ contains an alternative stretch $S^{C_{2}}_{i,j} \ne S^{H}_{i,j}$ and vice versa. Produce only those haplotypes from *C* for which every stretch in  contains at least one desired allele which is not present in the alternative stretch.

#### Heuristic H5c (consistent heuristic seed lots).

This heuristic is a stronger version of H5 that requires consistent improvement within all stretches towards a fixed haplotype of the corresponding ideotype chromosome.

For a homozygous ideotype, H5c degenerates to H5. To compute linkage phase ambiguities a heuristically constructed seed lot  is further extended to include all phase-known genotypes with the same allelic frequencies as any genotype already contained in . Heuristics H5 and H5c also provide an option to limit the number of simultaneous crossovers per chromosome.

Finally, heuristic H6 computes an approximate lower bound on the population size of any completed extension of a given partial schedule, based on the probabilities of those crossovers that are necessarily still required to obtain the ideotype.

#### Heuristic H6 (approximate population size bound).

From every chromosome *T* of the ideotype , with *n*_*T*_ loci, the set of desired stretches of size 2 is derived:

$$\mathcal{D}_{T} = \left\{S^{H}_{i,i+1}; H = T_{1} \vee T_{2}~\&~0 \le i < n_{T}-1\right\} $$

Only those stretches from $\mathcal {D}_{T}$ that do not occur in the respective chromosome of any parental genotype $G \in \mathcal {G}$ are retained. For each such stretch $S^{H}_{i,i+1}$ a crossover is necessarily required between loci *i* and *i*+1 to obtain the ideotype. Now, given a partial schedule, it is checked (for all chromosomes) which of the crucial stretches are not yet present in any genotype occurring in this schedule. The sum of the minimum population sizes required to obtain each of the corresponding crossovers is used as a lower bound for the increase in total population size of any completed extension of this schedule.

It might seem that heuristic H6 implements an exact bound but this is not guaranteed as Gene Stacker computes a joint population size when targeting multiple genotypes among the offspring of a shared seed lot (Additional file 1: Section 2). It is therefore possible that multiple crucial stretches are simultaneously obtained with a lower total cost. However, it is expected that this will rarely occur.

Several well-chosen combinations of heuristics provide tradeoffs between solution quality and execution time. Presets are named *best*, *better*, *default*, *faster* and *fastest*; ordered by the amount and restrictiveness of the applied heuristics. Full descriptions of the presets are included in the Additional file 1 (Section 5).

### Implementation and hardware

Gene Stacker is implemented in Java 7 and experiments have been performed on the UGent HPC infrastructure, using computing nodes with a 2.4 GHz quad-socket octa-core AMD Magny-Cours processor having a total of 32 cores and 64 GB RAM. Gene Stacker is freely available at http://genestacker.ugent.be; version 1.6 was used for all experiments. The website also contains user documentation and examples.

## Results and discussion

This section presents results of applying Gene Stacker to both generated and real stacking problems. First, some advantages of the extended DAG model are discussed. Then, the power of the applied optimization strategy in combination with the proposed heuristics is assessed. The section is concluded by providing some practical guidelines for users of Gene Stacker. Results are compared to those obtained by the method from [8], referred to as CANZAR^c^. This method minimizes the total population size, number of generations and total number of crossings. As minimizing the number of crossings is not explicitly considered as an objective in Gene Stacker, only schedules with the lowest total population size among those with the same number of generations, produced by CANZAR, were selected for comparison with Gene Stacker.

### Advantages of the extended model

Some advantages of the extended model are discussed here based on two constructed examples and a complex real stacking problem from cotton.

#### Constructed examples

Consider an example with two heterozygous parental genotypes *G*^1^,*G*^2^ and a heterozygous ideotype :

$$\begin{array}{@{}rcl@{}} G^{1} &=& \left[ \begin{array}{c} 0 \\ 1 \end{array} \right] \left[ \begin{array}{ccc} 0 & 0 & 0 \\ 0 & 0 & 1 \end{array} \right],\\ G^{2} &=& \left[ \begin{array}{c} 0 \\ 0 \end{array} \right] \left[ \begin{array}{ccc} 0 & 1 & 0 \\ 1 & 0 & 1 \end{array} \right],\\ \mathcal{I} &=& \left[ \begin{array}{c} 1 \\ 1 \end{array} \right] \left[ \begin{array}{ccc} 1 & 0 & 1 \\ 1 & 1 & 1 \end{array} \right]. \end{array} $$

The distance between the loci on the second chromosome is 31 and 42 cM, respectively. Five solutions were reported when running Gene Stacker in *default* mode, setting an overall success rate of *γ*=0.95 and a limit of 4 generations and 10% overall linkage phase ambiguity (Additional file 1: Figure S4).

**Figure 3:**
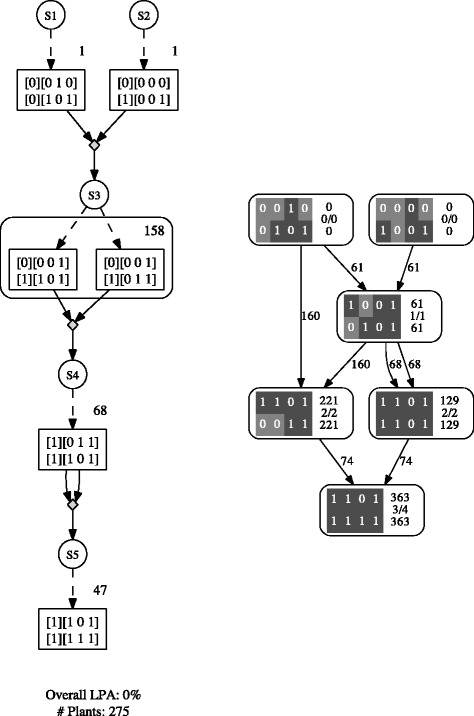
**Solutions for first constructed example.** (left) Best non-ambiguous three generation schedule obtained for the first constructed example when running Gene Stacker in *default* mode; (right) respective best three generation solution reported by CANZAR.

Figure 3 (left) shows the best non-ambiguous three generation schedule obtained by Gene Stacker, with a total population size of 275, as well as (right) the respective best three generation solution found by CANZAR, which has a higher total population size of 363. The leftmost target aimed for in the penultimate generation of the latter schedule has a linkage phase ambiguity of 23.1% while Gene Stacker’s solution is guaranteed to be non-ambiguous. Gene Stacker provides a way to avoid such high ambiguities by carefully monitoring them and considering ambiguity as an additional objective to be minimized.

This example also shows how computing joint population sizes when simultaneously targeting multiple genotypes among the offspring grown from a shared seed lot may significantly reduce the total population size (seed lot S3 in Figure 3). This approach enabled Gene Stacker to find an alternative schedule with a reduction of more than 24% in the total population size as compared to the schedule constructed by CANZAR.

Another advantage of representing plants and seed lots with distinct nodes is that (re)use of plants and seeds is differentiated. Gene Stacker only allows crossings with plants from the same generation, which is justified by the fact that almost all field crops flower only once, for a short time. Also for crops that flower multiple times or for a longer period (such as tomato), crossings with plants from distinct generations are usually not considered because of the high logistic impact. To repeatedly cross over multiple generations, the respective genotype has to be reproduced, for example by regrowing it from remaining seeds. In such case, the corresponding cost is accounted for. Note that this does not limit the flexibility of Gene Stacker’s model but ensures that the computed cost of the constructed schedules closely reflects plant breeding practice.

The next example has specifically been constructed to show the advantage of modelling multiple chromosomes. It consists of the following two parental genotypes and ideotype:

$$\begin{array}{@{}rcl@{}} G^{1} &=& \left[ \begin{array}{c} 0 \\ 0 \end{array} \right] \left[ \begin{array}{c} 0 \\ 1 \end{array} \right] \left[ \begin{array}{c} 0 \\ 1 \end{array} \right] \left[ \begin{array}{c} 0 \\ 1 \end{array} \right] \left[ \begin{array}{c} 0 \\ 1 \end{array} \right] \left[ \begin{array}{c} 0 \\ 1 \end{array} \right],~\\ G^{2} &=& \left[ \begin{array}{c} 0 \\ 1 \end{array} \right] \left[ \begin{array}{c} 0 \\ 1 \end{array} \right] \left[ \begin{array}{c} 0 \\ 1 \end{array} \right] \left[ \begin{array}{c} 0 \\ 1 \end{array} \right] \left[ \begin{array}{c} 0 \\ 1 \end{array} \right] \left[ \begin{array}{c} 0 \\ 0 \end{array} \right],\\ \mathcal{I} &=& \left[ \begin{array}{c} 0 \\ 1 \end{array} \right] \left[ \begin{array}{c} 0 \\ 1 \end{array} \right] \left[ \begin{array}{c} 0 \\ 1 \end{array} \right] \left[ \begin{array}{c} 0 \\ 1 \end{array} \right] \left[ \begin{array}{c} 0 \\ 1 \end{array} \right] \left[ \begin{array}{c} 0 \\ 1 \end{array} \right]. \end{array} $$

**Figure 4:**
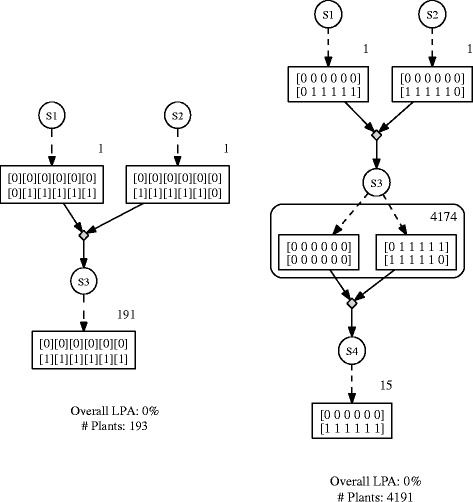
**Solutions for second constructed example.** (left) Best solution obtained for the second constructed example when explicitly modelling multiple chromosomes; (right) best solution found when combining all loci on one artificial chromosome, where a crossover rate of 0.5 is specified between pairs of consecutive loci that actually reside on different chromosomes (in this example, all loci).

A genetic map is not required as each chromosome contains one locus only. Running Gene Stacker with any preset and *γ*=0.95 resulted in the schedule from Figure 4 (left) which performs a single crossing. It is possible to immediately obtain the ideotype from this crossing because the order of haplotypes within a chromosome is arbitrary. This is taken into account when computing the probability of observing a genotype among the offspring (Additional file 1: Section 1). In contrast, previous methods used a single chromosome and specified a recombination rate of 0.5 between loci that actually reside on different chromosomes. This requires to arbitrarily fix an order of haplotypes in each actual chromosome and artificially increases the complexity of the problem. Figure 4 (right) shows Gene Stacker’s solution for the same example when combining all loci on a single artificial chromosome. This schedule is significantly worse: it has an additional generation and a much higher total population size. Although this example was specifically constructed and is somewhat extreme in the sense that it has six loci on six different chromosomes, it clearly shows the general benefits of explicitly modelling multiple chromosomes.

#### Dealing with tight constraints

Tight constraints might apply for specific crops. For example, cotton plants can be used for two crossings only (or one selfing) and each crossing yields a small amount of about 250 seeds. This makes it more difficult to find good crossing schedules within the constraints. With the extended model such important constraints can easily be taken into account. Crossings are performed multiple times if necessary to provide a sufficient amount of seeds, where sometimes several duplicates of the same genotype are needed to be able to make all crossings. Population sizes are computed in such way that at least the required number of duplicates of each targeted genotype is expected among the offspring (Additional file 1: Section 2).

An example from cotton is considered with 6 parental genotypes, 11 loci spread across 5 chromosomes and a heterozygous ideotype (full description in Additional file 1: Section 7). An overall success rate of *γ*=0.95 was used and the number of generations, number of plants per generation and overall linkage phase ambiguity were limited to 5, 5000 and 10%, respectively. A time limit of 24 hours was applied. The number of crossings per plant and seeds obtained per crossing were set to 2 and 250, respectively, to precisely reflect the tight constraints of cotton breeding.

Running Gene Stacker with preset *fastest* took 2 hours and 15 minutes to complete and reported 4 solutions with 3–5 generations, a total population size of 7256–1077 and an overall linkage phase ambiguity of 0–3.14% (Additional file 1: Figures S5–S8). All other presets ran out of memory (64 GB). When restricting the number of generations to 4 instead of 5, preset *faster* reported a different solution with 4 generations that has a lower total population size (1400) than the respective schedule found by preset *fastest* (1534) before being interrupted when the time limit of 24 hours had been exceeded (Additional file 1: Figure S9). All solutions contain at least one crossing which is performed multiple times and/or a genotype of which multiple duplicates are selected. It was not possible to obtain solutions within the constraints using CANZAR as this method does not provide a way to accurately impose and work around these constraints.

### Optimization power and heuristics

We first explore the limits of the optimization strategy and the power gained by applying additional heuristics, based on experiments with a large number of randomly generated problem instances. Then, the obtained quality-runtime tradeoff is assessed for various complex, real stacking problems.

#### Limits of the optimization strategy

Experiments have been performed with a variety of 240 randomly generated stacking problems; 120 with a homozygous ideotype and 120 with a heterozygous ideotype. All instances have 4–14 loci, taking steps of two, and 20 instances were created for every number of loci and for both types of ideotype. Each instance has been independently generated by

(i) picking a random number of 1–8 chromosomes, limited by the number of loci;
(ii) randomly assigning each locus to one of the available chromosomes, with a minimum of 1 locus per chromosome;
(iii) setting a random distance of 1–50 cM between pairs of consecutive loci on the same chromosome;
(iv) randomly creating 2–8 parental genotypes, where each allele is set to 1 or 0 with equal probability; and
(v) generating a random ideotype.

The haplotypes of the ideotype’s chromosomes were created by copying alleles from one of both haplotypes of the respective chromosome of a randomly picked parental genotype (independently for every locus). To obtain a homozygous ideotype, one haplotype is created for each chromosome and included twice. For heterozygous ideotypes, two independent haplotypes are created and combined for every chromosome.

**Figure 5:**
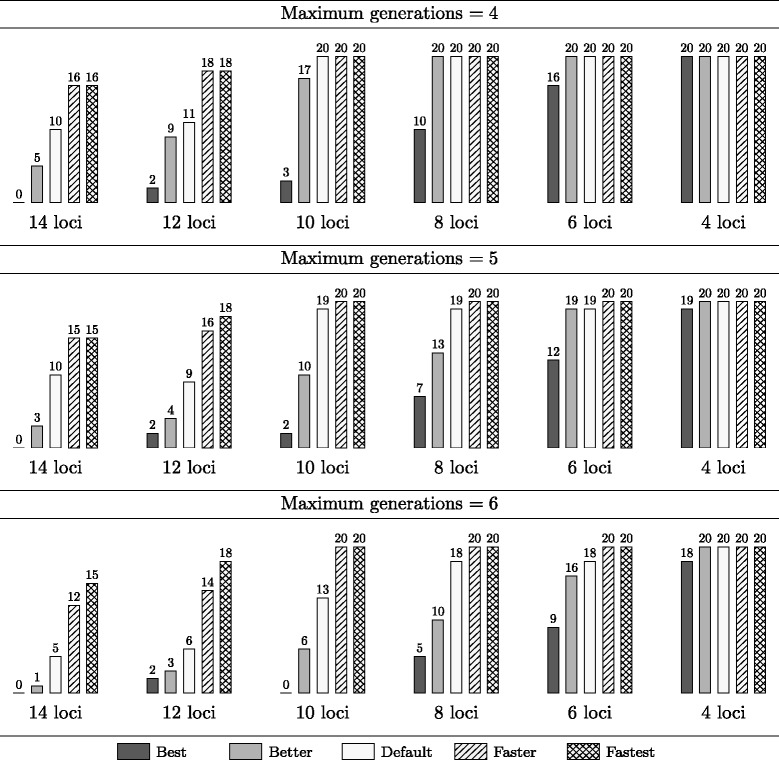
**Results for random instances with a homozygous ideotype.** This figure indicates the number of randomly generated instances with a *homozygous* ideotype for which the different presets of Gene Stacker completed within the applied time limit of 24 hours. Experiments were repeated with a maximum of 4–6 generations. Instances have 4–14 loci spread across 1–8 chromosomes and 2–8 parental genotypes. In total, 20 instances were generated for each number of loci.

Figure 5 shows results of running each preset of Gene Stacker on the 120 instances with a homozygous ideotype. All experiments have been repeated with a maximum of 4, 5 and 6 generations, and a runtime limit of 24 hours has been applied, together with an overall success rate of *γ*=0.95 and a maximum of 10000 plants per generation, 4 crossings per plant, 5000 obtained seeds per crossing and 20% overall linkage phase ambiguity. For every combination of the maximum number of generations (rows), the number of loci (columns) and the applied preset (bars) it is reported for how many out of 20 instances Gene Stacker completed within the time limit of 24 hours.

Without applying any heuristics (preset *best*), Gene Stacker solves only 42.5%, 35% and 28.34% of all instances when limiting the number of generations to 4, 5 and 6, respectively. Interestingly, solutions are obtained for about 95% of all instances when applying all heuristics (preset *fastest*) regardless of the limit on the number of generations. As expected (and desired), the power of the other presets (*better*, *default*, *faster*) lies somewhere in between. The problem complexity obviously increases with the number of loci as well as the maximum number of generations. Without any heuristics, Gene Stacker solved almost no problems with more than 8 loci: solutions were obtained for less than half of the instances when the number of loci exceeded 8, 6 and 4 with a limit of 4, 5 and 6 generations, respectively. Yet, Gene Stacker can cope with many more complex problems with up to at least 14 loci using the proposed heuristics. Of course, these heuristics may yield worse Pareto frontier approximations, so it is preferred only to enable them if necessary to find solutions within reasonable time. In this way, the heuristics offer a convenient quality-runtime tradeoff and allow to obtain (approximate) solutions for more complex problems.

**Figure 6:**
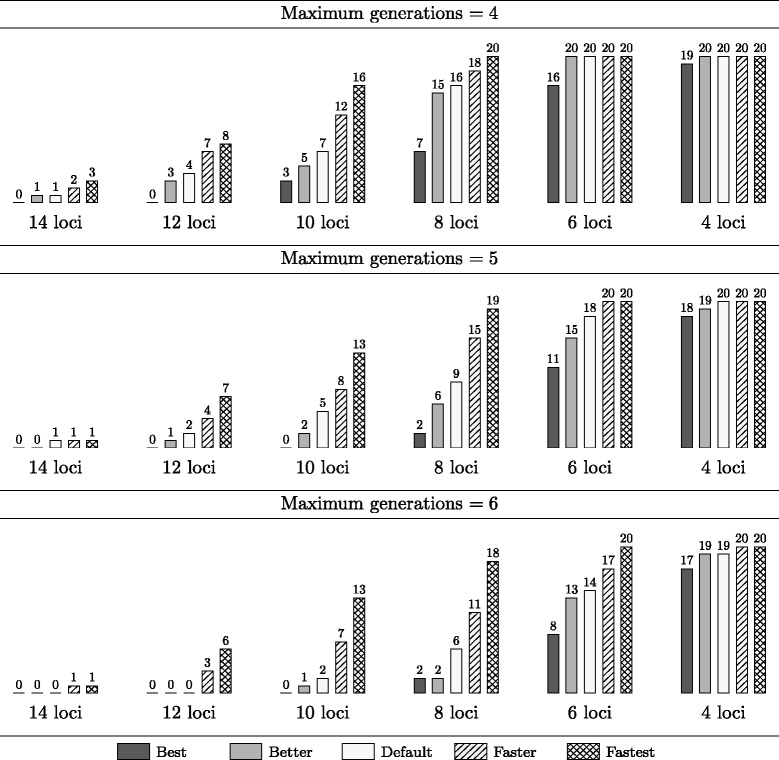
**Results for random instances with a heterozygous ideotype.** This figure indicates the number of randomly generated instances with a *heterozygous* ideotype for which the different presets of Gene Stacker completed within the applied time limit of 24 hours. Experiments were repeated with a maximum of 4–6 generations. Instances have 4–14 loci spread across 1–8 chromosomes and 2–8 parental genotypes. In total, 20 instances were generated for each number of loci.

Figure 6 shows similar results for the 120 instances with a heterozygous ideotype. It is clear that these are generally more complex as significantly fewer instances were solved within the time limit compared to the results from Figure 5. This may be explained from the fact that each heterozygous chromosome in the ideotype contains two different target haplotypes, i.e. two competing goals, that have to be obtained simultaneously. Also, the heuristics are less effective for heterozygous ideotypes. For example, improvement towards any of both haplotypes of a heterozygous ideotype chromosome is rewarded; therefore, heuristics based on such improvement are less powerful in case of two distinct target haplotypes in a single chromosome.

Without applying any heuristics, Gene Stacker now solves 22.5–37.5% of all instances for a varying limit on the number of generations. Less than half of the instances were solved when the number of loci exceeded 4–6. When all heuristics are enabled, solutions are obtained for 65–72.5% of the instances (for less than half of the instances when exceeding 10 loci). Although the currently proposed heuristics are clearly less powerful when aiming for a heterozygous ideotype, they allowed to find solutions for many complex problems with up to 10 loci. Nevertheless, the challenge remains to develop better heuristics in this respect.

We conclude that the applied optimization strategy can effectively be used to find solutions for a wide range of stacking problems. Without extra heuristics, some smaller problems with 4–8 or 4–6 loci in case of a homozygous or heterozygous ideotype, respectively, can already be tackled (depending on the maximum number of generations). To deal with more complex problems, additional heuristics are required. The proposed heuristics allow to obtain (approximate) solutions for problems with up to at least 10–14 loci.

#### Quality-runtime tradeoff

The quality-runtime tradeoff obtained by applying different combinations of heuristics is assessed here for real stacking problems from tomato and rice (full specification in Additional file 1: Section 7). For all experiments, an overall success rate of *γ*=0.95 was set and the number of generations and plants per generation were restricted to 5 and 5000, respectively. The amount of seeds produced per crossing and maximum number of crossings per plant were set to reflect the specific properties of each crop. Approximated Pareto frontiers in terms of the total population size and number of generations are reported (only schedules with zero linkage phase ambiguity were selected).

First, experiments were performed with two stacking problems from tomato. Both consist of the same 4 parental genotypes with 8 loci spread across 6 chromosomes. The first example (Tomato-1) has a homozygous ideotype while the second example (Tomato-2) has a heterozygous ideotype. Tomatoes can easily be crossed several dozens of times and every crossing yields a large number of seeds: the maximum number of crossings per plant and the amount of seeds obtained from one crossing were set to 24 and 20000, respectively. A time limit of 12 hours was imposed, after which the algorithms were interrupted and the solutions found until then were inspected.

**Figure 7:**
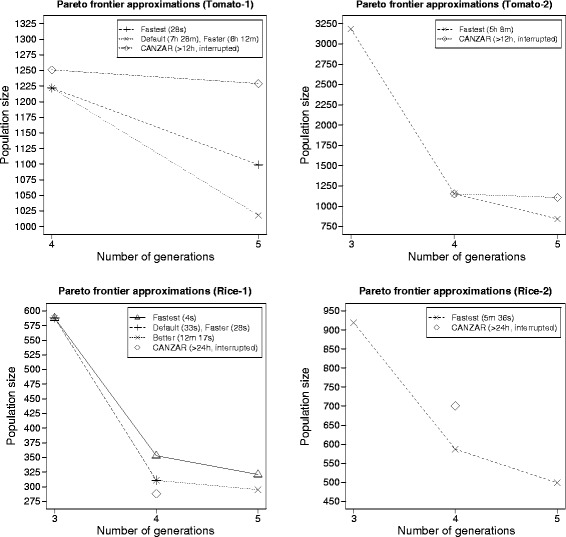
**Pareto frontier approximations of real stacking problems from tomato and rice.** (top left) First example from tomato (Tomato-1), homozygous ideotype; (top right) second example from tomato (Tomato-2), heterozygous ideotype; (bottom left) first example from rice (Rice-1), homozygous ideotype; (bottom right) second example from rice (Rice-2), heterozygous ideotype. Full descriptions of the examples are provided in the Additional file 1: Section 7.

Figure 7 (top left) shows the Pareto frontier approximations obtained by applying Gene Stacker with presets *default*, *faster* and *fastest* as well as CANZAR to Tomato-1. Gene Stacker and CANZAR obtained exactly the same schedule with 4 generations. The small difference in the reported population size is explained by the fact that both methods follow a slightly different approach to derive a success rate per targeted genotype (*γ*^′^) from the desired overall success rate (*γ*). Solutions with 5 generations were also found. Those reported by Gene Stacker have a lower population size compared to the one obtained by CANZAR, even when applying preset *fastest* which completes after only 28 seconds. Presets *default* and *faster* reported exactly the same solutions and the 5 generation schedule found here improves over the respective schedule obtained by preset *fastest*. Yet, these two presets took significantly more time (ca. 6–8 hours). This shows how the proposed heuristics provide tradeoffs between solution quality and execution time and that they are capable of finding good solutions for a complex, realistic problem within reasonable time. CANZAR was interrupted when exceeding the time limit of 12 hours.

Similar results for Tomato-2 are presented in Figure 7 (top right) where only preset *fastest* has been applied since the other presets ran out of memory (64 GB). Gene Stacker completed in about 5 hours while CANZAR was interrupted when the time limit had expired. Three solutions were reported by Gene Stacker with 3–5 generations and CANZAR obtained 2 solutions with 4–5 generations. The 4 generation schedules reported by both methods slightly differ but have approximately the same total population size. Conversely, Gene Stacker found a somewhat better schedule with 5 generations and an additional solution with only 3 generations. The difference in runtime, as compared to Tomato-1, and the fact that all other presets ran out of memory again confirm that with the current heuristics it is more difficult to solve stacking problems with a heterozygous ideotype. Yet, the heuristics made it possible to find 3 good solutions within a few hours, using a transparent optimization strategy.

Results were also obtained for two examples from rice. Both consist of the same 8 parental genotypes with 10 loci spread across 6 chromosomes. The first example (Rice-1) has a homozygous ideotype while the second example (Rice-2) has a heterozygous ideotype. About 300 seeds are obtained from each crossing and rice plants can be crossed no more than 5 times. For these examples, a time limit of 24 hours was set.

Figure 7 (bottom left) shows the Pareto frontier approximations obtained by applying Gene Stacker with presets *better*, *default*, *faster* and *fastest* as well as CANZAR to Rice-1. Preset *fastest* completed after only 4 seconds and reported three solutions with 3–5 generations. Presets *default* and *faster* terminated after about 30 seconds and found a better schedule that dominates both the 4 and 5 generation schedules obtained by preset *fastest*. Preset *better* completed after about 12 minutes and found an additional 5 generation schedule with a slightly lower total population size. This again shows how the heuristics offer a convenient quality-runtime tradeoff. CANZAR did not complete within the time limit of 24 hours but was able to obtain a single schedule with 4 generations that dominates all 4 and 5 generation schedules obtained by Gene Stacker. It is inevitable that the heuristics sometimes make wrong decisions in which case valuable parts of the search space may not have been explored. In this specific example, heuristic H0 (included in all presets except preset *best*) removed a parental genotype that is needed to find the better schedule obtained by CANZAR. Still, results are quite close to those of CANZAR, especially when applying presets *faster*, *default* or *better*, a significant speedup is obtained and an additional solution with only 3 generations is found.

Similar results for Rice-2 are shown in Figure 7 (bottom right) where only preset *fastest* has been applied as the other presets either ran out of memory or did not find any solutions within the time limit. Gene Stacker completed after 5–6 minutes while CANZAR was interrupted after exceeding the time limit of 24 hours. Three solutions were reported by Gene Stacker, with 3–5 generations. CANZAR found a single solution with 4 generations and a higher population size than the respective schedule obtained by Gene Stacker. Again, the runtime and memory footprint of Gene Stacker is significantly higher for this problem with a heterozygous ideotype as compared to Rice-1 which has a homozygous ideotype. Yet, preset *fastest* outperforms CANZAR and is able to provide a valuable approximation of the Pareto frontier within a few minutes.

### Practical guidelines

Based on our findings we propose the following practical guidelines for using Gene Stacker. Best is to first try the default settings, specifying the required parameters (maximum number of generations and overall success rate) and those constraints that are important for the specific application (such as the number of seeds produced from a crossing and maximum number of crossings per plant) with a reasonable runtime limit (e.g. 24 hours). If Gene Stacker is too slow or requires too much memory, consider setting additional or tighter constraints (e.g. maximum plants per generation, maximum overall linkage phase ambiguity,...) and/or using preset *faster* or *fastest*. The latter may yield worse solutions which should be avoided when possible. In case the default setting is more than fast enough consider running presets *better* and *best* as well to check whether this produces better schedules, as the heuristics might have missed something. Usually, differences between the latter presets and the default setting are very small (if any) except for the runtime which is significantly increased.

In case QTL (quantitative trait locus) intervals need to be stacked one can use flanking markers to delimit the target locus. The Tomato-1 problem (Additional file 1: Section 7) is a case in point. On the sixth chromosome, a small region of 10 cM has been identified in which a target gene is located. In this setting it is advised to make sure that the required haplotype is present in at least one of the parents, and to verify it is maintained throughout the crossing scheme. There always remains a small risk of a double cross-over within the interval in a single generation which one can either ignore or monitor by saturating the interval with additional markers. More details and practical examples are given at http://genestacker.ugent.be.

## Conclusions

The proposed transparent, flexible and easily extensible approach to marker-assisted gene pyramiding was confirmed to be feasible in combination with heuristics to address realistic, complex stacking problems with up to at least 10–14 loci, while taking into account important breeding constraints. Carefully designed heuristics even allow to find better or additional solutions within reasonable time compared to previous methods. The proposed heuristics are certainly not perfect nor complete. For example, they are less effective for problems with a heterozygous ideotype. Future work may include the design of additional or improved heuristics as well as extension of the ideas applied in Gene Stacker for a more general plant breeding context that also addresses complex traits and conservation of genetic background.

## Availability of supporting data

The data set(s) supporting the results of this article is(are) included within the article (and its additional file(s)).

## Endnotes

^a^ See http://cplex.com.

^b^ The term ‘homozygous genotype’ is used to indicate that all considered loci are homozygous; this does not say anything about the remaining loci in the full DNA and should for example not be confused with homozygous inbred lines.

^c^ Experiments with CANZAR were run on the SURFsara Lisa computing system (https://www.surfsara.nl/systems/lisa/description) by the authors of this method.

## Additional file

Additional file 1 **Supplementary material.** PDF file with supplementary information such as formulas, algorithm details and additional results.

## Abbreviations

MIP: Mixed integer programming
DAG: Directed acyclic graph
LPA: Linkage phase ambiguity
QTL: Quantitative trait locus
