# Supplementary material for: Heuristic exploitation of genetic structure in marker-assisted gene pyramiding problems
Source: BMC Genet. 2015 Jan 30;16:2. doi: 10.1186/s12863-014-0154-z (PMC4332449; doi:10.1186/s12863-014-0154-z)
Supplement: Supplementary file 1 — Supplementary material. PDF file with supplementary information such as formulas, algorithm details and additional results. [file 12863_2014_154_MOESM1_ESM.pdf]

Supplementary Material

for

Heuristic exploitation of genetic structure in marker-assisted gene pyramiding problems

1 Recombination rates

When crossing two genotypes P and Q a number of possible genotypes can occur among the offspring due to recombination of alleles, each with a certain probability. This section describes how to compute these probabilities based on the recombination rates $r_{i,p,q}$ that indicate the expected frequency of crossovers between the pth and qth loci of the ith chromosome and which are inferred from the genetic map.

First, take the ith chromosome P_i of genotype P and any haplotype H_i with the same number of loci as P_i. Suppose that P_i contains l heterozygous loci with ordered indices $s = (\nu_1, \ldots, \nu_l)$. Then, the probability $Pr[P_i \rightarrow H_i]$ that haplotype H_i is produced from chromosome P_i is computed as follows [1]:

- If H_i contains at least one allele which does not occur at the respective locus in P_i then $Pr[P_i \rightarrow H_i] = 0$, i.e. it is impossible that H_i will be produced from P_i.
- Else, if s is empty (all loci are homozygous), $Pr[P_i \rightarrow H_i] = 1$.
- Else

$$Pr[P_i \rightarrow H_i] = \frac{1}{2} \prod_{j=1}^{l-1} \begin{cases} r_{i,\nu_j,\nu_{j+1}} & \text{in case of a crossover} \\ & \text{between loci } \nu_j \text{ and } \nu_{j+1} \\ 1 - r_{i,\nu_j,\nu_{j+1}} & \text{otherwise} \end{cases}$$

where there has been a crossover between loci ν_j and ν_{j+1} if

$$H_i(\nu_j) = P_{i,1}(\nu_j) \quad \wedge \quad H_i(\nu_{j+1}) = P_{i,2}(\nu_{j+1}), \text{ or}$$
$$H_i(\nu_j) = P_{i,2}(\nu_j) \quad \wedge \quad H_i(\nu_{j+1}) = P_{i,1}(\nu_{j+1}).$$

The factor of 1/2 is introduced because every sequence of crossovers defines two complementary haplotypes which are inherited with equal probability.

Now, take any chromosome G_i with the same number of loci as the ith chromosomes P_i and Q_i of both parents P and Q. The probability $Pr[P_i, Q_i \rightarrow G_i]$ that chromosomes P_i and Q_i will produce haplotypes which together form a chromosome G_i is computed as follows:

$$Pr[P_i, Q_i \rightarrow G_i] = \begin{cases} Pr[P_i \rightarrow G_{i,1}] \cdot Pr[Q_i \rightarrow G_{i,2}] & \text{if } G_{i,1} = G_{i,2} \\ Pr[P_i \rightarrow G_{i,1}] \cdot Pr[Q_i \rightarrow G_{i,2}] & \text{if } G_{i,1} \neq G_{i,2} \\ \quad + Pr[P_i \rightarrow G_{i,2}] \cdot Pr[Q_i \rightarrow G_{i,1}]. & \end{cases}$$

The second case accounts for the fact that the haplotypes might swap their originating parents. Gene Stacker explicitly models multiple chromosomes: the probability $Pr[P, Q \rightarrow G]$ of obtaining the entire phase-known genotype G from crossing the phase-known parents P and Q, with k chromosomes, is computed by multiplying the independent chromosome probabilities:

$$Pr[P, Q \rightarrow G] = \prod_{i=1}^{k} Pr[P_i, Q_i \rightarrow G_i].$$

Note that this will account for up to 2^k identical phase-known genotypes G depending on how many haplotype pairs might swap their originating parents.

2 Joint population sizes

Sometimes several different genotypes or multiple occurrences of the same genotype are simultaneously targeted among offspring grown from a shared seed lot. In such case it is possible to compute

a joint population size, i.e. the number of offspring that needs to be generated so that at least the number of desired occurrences of each targeted genotype are expected to be obtained. The same individual and overall success rates can still be guaranteed, but the computed joint population size is often much smaller than the sum of the population sizes required to obtain each targeted genotype individually. This procedure may therefore significantly reduce the total population size.

Suppose that m distinct phase-known genotypes $G_1, \ldots, G_m$ are targeted among the offspring, where $f_1, \ldots, f_m$ occurrences of the respective targets are desired, with $f_i \geq 1, \forall i = 1, \ldots, m$ and $\sum_{i=1}^{m} f_i = f$. The joint population size N is then calculated as follows:

1. Compute the population sizes N_i required to obtain each target G_i individually using formula (1) from the main article.

2. Set
$$N = \max\{N_i | i = 1, \ldots, m\}$$
and compute the joint success probability (see below)
$$P = Pr[|G_1| \geq f_1 \ \& \ \cdots \ \& \ |G_m| \geq f_m; N].$$

3. If $P < (\gamma')^f$, adjust N using binary search in the interval
$$I = [\max\{N_i | i = 1, \ldots, m\}, \sum_{i=1}^{m} (f_i \cdot N_i)]$$
to find the smallest $N \in I$ for which $P \geq (\gamma')^f$.

Formulas to compute $Pr[|G_1| \geq f_1 \ \& \ \cdots \ \& \ |G_m| \geq f_m; N]$ are stated below. Taking the maximum in step 2 ensures a success rate of at least γ' for every target individually. Subsequently, step 3 further increases this initial estimate of the joint population size to guarantee a joint success rate of at least $(\gamma')^f$ so that this joint trial with f targets still contributes a factor of $(\gamma')^f$ to the overall success rate − just as if f independent Bernoulli trials would have been performed. Such independent trials would require a population size of $\sum_{i=1}^{m} (f_i \cdot N_i)$ to obtain all targets, which is the upper bound of I. By considering a joint trial, the total population size can be significantly reduced while both the same individual and overall success rates are still guaranteed. Experiments showed that in practice step 3 rarely has to be executed, leading to a significant reduction in population size with almost no computational overhead.

The joint success probability $Pr[|G_1| \geq f_1 \ \& \ \cdots \ \& \ |G_m| \geq f_m; N]$ of obtaining each targeted phase-known genotype G_i at least f_i times, when growing N plants in total, is computed as:

$$
\begin{aligned}
Pr[|G_1| \geq f_1 \ \& \ \cdots \ \& \ |G_m| \geq f_m; N] &= 1 - \neg Pr[|G_1| \geq f_1 \ \& \ \cdots \ \& \ |G_m| \geq f_m; N] \\
&= 1 - Pr[|G_1| \leq (f_1 - 1) \ \vee \ \cdots \ \vee \ |G_m| \leq (f_m - 1); N] \\
&= 1 - \sum_{i_1 \in [1,m]} Pr[|G_{i_1}| \leq (f_{i_1} - 1); N] \\
&+ \sum_{\substack{(i_1,i_2) \in [1,m]^2 \\ i_1 < i_2}} Pr[|G_{i_1}| \leq (f_{i_1} - 1) \ \& \ |G_{i_2}| \leq (f_{i_2} - 1); N] \\
&- \sum_{\substack{(i_1,i_2,i_3) \in [1,m]^3 \\ i_1 < i_2 < i_3}} Pr[|G_{i_1}| \leq (f_{i_1} - 1) \ \& \ |G_{i_2}| \leq (f_{i_2} - 1) \\
&\qquad \& \ |G_{i_3}| \leq (f_{i_3} - 1); N] \\
&+ \cdots \\
&\cdots \\
&+ (-1)^m \ Pr[|G_1| \leq (f_1 - 1) \ \& \ \cdots \ \& \ |G_m| \leq (f_m - 1); N]
\end{aligned}
$$

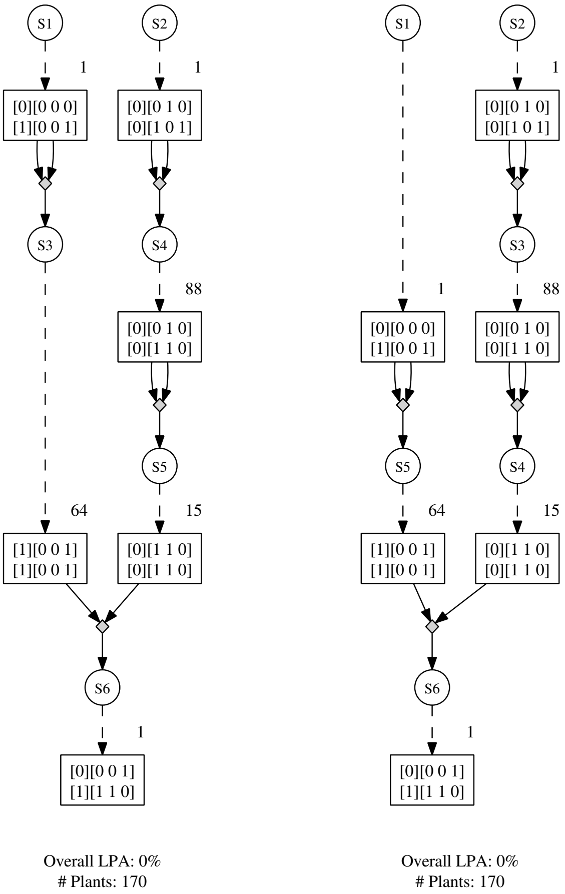

Figure S1: Alternatives of the same schedule, obtained from a different alignment of generations, which are equally good in terms of the considered objectives (number of generations, total population size and overall linkage phase ambiguity). Both alternatives are retained and will be considered for further extension.

with

$$
\begin{aligned}
& Pr[|G_{i_1}| \leq f_{i_1} \ \& \ \cdots \ \& \ |G_{i_k}| \leq f_{i_k}; N] \\
= \quad & \sum_{n_1=0}^{f_{i_1}} \cdots \sum_{n_k=0}^{f_{i_k}} \frac{N \cdot (N-1) \cdots (N - n_1 - \cdots - n_k + 1)}{n_1! \cdots n_k!} p_{G_{i_1}}^{n_1} \cdots p_{G_{i_k}}^{n_k} \cdot (1 - p_{G_{i_1}} - \cdots - p_{G_{i_k}})^{N - n_1 - \cdots - n_k}
\end{aligned}
$$

where p_{G_i} is the probability of observing G_i among the offspring. These formulas follow from the multinomial probability distribution. The joint success probability is computed through its complement as the f_i's are expected to be small – often they are all equal to 1 – while N is expected to be much larger. Also, m is expected to be relatively small. Therefore, a direct computation would require to sum over significantly more terms, compared to this computation through the complement.

3 Alignment of generations

When combining two partial schedules through a crossing of their final plants, the generations of these schedules can be aligned in different ways. Figure S1 shows two equally good alternatives of

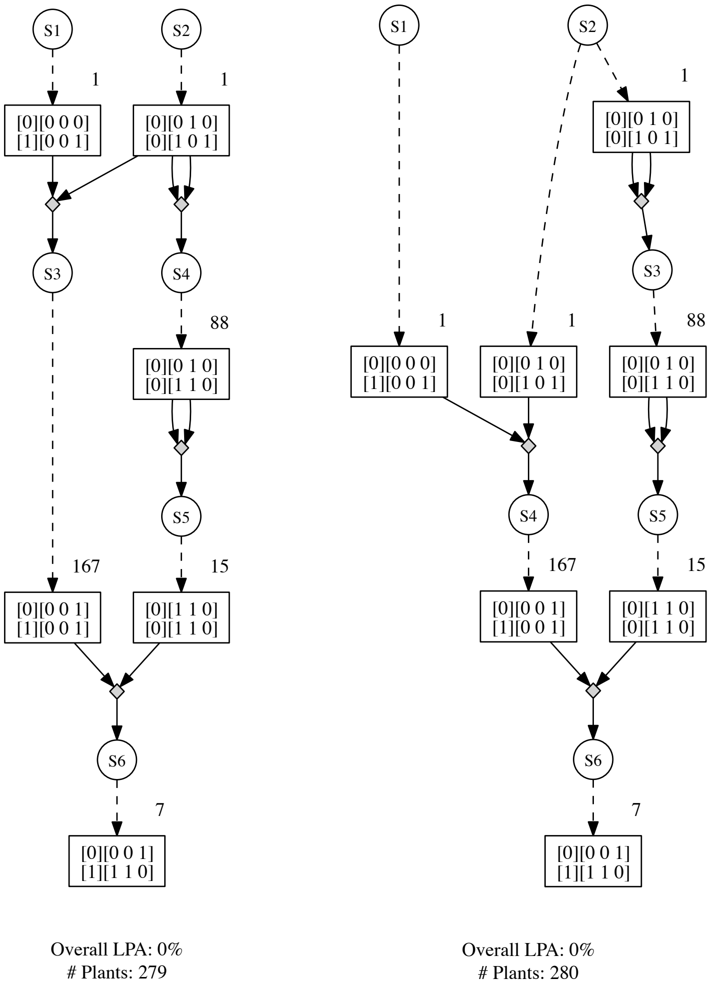

Figure S2: This figure shows two alternative alignments of generations in a crossing schedule, where the left alignment is preferred over the right alignment since it has a lower total population size, the same number of generations and the same overall linkage phase ambiguity. By performing the crossing that creates seed lot S3 in the first generation (left), one parent plant can be reused, while this plant has to be regrown if the crossing is postponed to the second generation (right). The left alignment is retained, but the right alignment is (greedily) discarded.

the same schedule, obtained from a different alignment of the generations of the smaller schedules from which the larger schedule was created. Both alignments are retained and will be considered for further extension. In contrast, Figure S2 shows two alternative alignments of generations in a crossing schedule where one alignment is greedily discarded (right) because it is dominated by the other alignment (left) in terms of the objectives (number of generations, total population size and overall linkage phase ambiguity).

4 Detailed algorithm

Figure S3 provides a detailed outline of the Gene Stacker algorithm. The input consists of a set of parental genotypes $\mathcal{G}$, the desired ideotype $\mathcal{I}$ and a genetic map $\mathcal{M}$. As output, an approximated Pareto frontier $\mathcal{F}$ is produced. The queue $\mathcal{Q}$ contains those schedules that still have to be extended and the algorithm iteratively dequeues partial schedules C from $\mathcal{Q}$ to create larger schedules C_{new}

```
function GeneStacker(𝒢, ℐ, ℳ)
    𝒬 ← [ ]                                                     ▷ queue containing schedules to be extended
    𝒫 ← [ ]                                                     ▷ previously extended schedules
    ℱ ← [ ]                                                     ▷ current Pareto frontier
    for all parental genotypes P ∈ 𝒢 do
        add minimal schedule growing P to 𝒬                     ▷ add minimal schedules to queue
    end for
    while 𝒬 not empty do
        C ← dequeue element from 𝒬                              ▷ schedule to be extended (≥ 1 alternatives C[i])
        𝒮 ← Self(final plant from C, ℳ)                         ▷ compute seed lot obtained by selfing
        for all genotypes G ∈ 𝒮 do                              ▷ consider each genotype in 𝒮 as next target
            C_new ← [ ]
            for i = 0, . . . , |C| − 1 do                       ▷ consider all alternatives of C
                C_new[i] ← attach selfing, 𝒮 and G to C[i]      ▷ extend C[i] to create C_new[i]
            end for
            RegisterSchedule(C_new, ℐ, ℱ, 𝒬)                    ▷ register new schedule (all alternatives)
        end for
        for all C′ ∈ 𝒫 do                                       ▷ combine with previous schedules
            A ← [ ]
            for i = 0, . . . , |C| − 1 do                       ▷ align alternatives of C and C′ (pairwise)
                for j = 0, . . . , |C′| − 1 do
                    B ← Align(C[i], C′[j])                      ▷ align alternatives C[i] and C′[j]
                    for all B′ ∈ B do                           ▷ store constructed alignments
                        add B′ to A
                    end for
                end for
            end for
            FilterAlignments(A)                                 ▷ remove non Pareto optimal alignments
            𝒮 ← Cross(final plant from C, final plant from C′, ℳ)   ▷ compute seed lot obtained by crossing
            for all genotypes G ∈ 𝒮 do                          ▷ consider each genotype in 𝒮 as next target
                C_new ← [ ]
                for i = 0, . . . , |A| − 1 do                   ▷ consider all retained alignments
                    C_new[i] ← attach crossing, 𝒮 and G to A[i] ▷ extend A[i] to create alternative C_new[i]
                end for
                RegisterSchedule(C_new, ℐ, ℱ, 𝒬)                ▷ register new schedule (all alternatives)
            end for
        end for
        Add C to 𝒫                                              ▷ add to list of already extended schedules
    end while
    return ℱ                                                    ▷ return final Pareto frontier approximation
end function

function RegisterSchedule(C_new, ℐ, ℱ, 𝒬)
    for i = 0, . . . , |C_new| − 1 do                           ▷ consider all alternatives of C_new
        ResolveDepletedSeedLots(C_new[i])                       ▷ resolve any depleted seed lots
        if ideotype ℐ obtained and constraints satisfied then
            Update ℱ with new solution C_new[i]                 ▷ update Pareto frontier
            Remove C_new[i] from C_new                          ▷ discard alternative (complete)
        else if Prune(C_new) then                               ▷ check (heuristic) pruning criteria
            Remove C_new[i] from C_new                          ▷ discard alternative (pruned)
        end if
    end for
    if |C_new| > 0 then                                         ▷ check if any alternatives remain
        Add C_new to 𝒬                                          ▷ queue schedule for further extension
    end if
end function
```

Figure S3: Detailed outline of the Gene Stacker algorithm. The input consists of a set of parental genotypes $\mathcal{G}$, the ideotype $\mathcal{I}$ and the genetic map $\mathcal{M}$. Crossing schedules are iteratively extended to approximate the Pareto frontier of solutions with minimum number of generations, total population size and overall linkage phase ambiguity.

by (a) selfing the final plant of C; and (b) combining C with each previously extended schedule C' through a crossing of the final plants of both schedules. More precisely, every element $C \in \mathcal{Q}$ consists of a series of $a \geq 1$ alternatives $C[0], \ldots, C[a-1]$ of the same schedule. These alternatives arise because there are several ways to align or interleave the generations of two smaller schedules C and C' when combining them into a larger schedule C_{new}: each generation of C_{new} either contains one single generation from C or C', or consists of the alignment of two generations; one from each of the smaller schedules (see previous section for examples).

Whenever a crossing or selfing is performed to extend a schedule, the corresponding seed lot $\mathcal{S}$ is constructed by (a) inferring all possible haplotypes that can be produced from each chromosome of both parents; (b) creating all pairwise combinations, per chromosome, of haplotypes produced by both parents; and (c) making all combinations of the obtained chromosomes. This yields the set of possible offspring. During generation, the corresponding probabilities and linkage phase ambiguities are computed.

After selfing the final plant of a partial schedule C, Gene Stacker considers each genotype G in the constructed seed lot $\mathcal{S}$ to be fixed as a possible next target. For each genotype G, the alternatives of a larger schedule C_{new} are created by attaching G to each alternative $C[i]$ of C.

To combine two partial schedules C and C' through a crossing of their final plants, Gene Stacker first creates all alignments A of all pairs of alternatives $C[i]$ and $C'[j]$. Alignments are constructed in a *bottom-up* fashion: first, the new crossing node is created, joining the parent plants, and then the alignments are further completed by repeatedly inserting the previous generation from either $C[i]$, $C'[j]$ or both smaller schedules. Plant nodes and seed lot nodes occurring in both smaller schedules which end up being aligned in the same generation of the new schedule are dynamically reused. Of all constructed alignments within the constraints, only Pareto optimal alignments are retained (in terms of the objectives of the main optimization problem); other alignments are greedily discarded. Then, for every genotype G in $\mathcal{S}$, the alternatives of a larger schedule C_{new} are created by attaching G as the next target to each retained alignment $A[i]$.

For each alternative of every newly created schedule C_{new} it is checked whether there are any depleted seed lots, i.e. seed lots from which more seeds are taken than the amount provided by the performed crossing(s). In such case, Gene Stacker indicates that the crossing should be performed multiple times to provide additional seeds. For this, it may be necessary to have several duplicates of the crossed genotypes, taking into account the number of crossings that can be performed with a single plant. This affects the population sizes and may introduce new depleted seed lots; therefore, this process is repeated until all depleted seed lots have been resolved.

If the ideotype $\mathcal{I}$ is obtained, each alternative $C_{new}[i]$ for which all constraints are satisfied is registered in the Pareto frontier $\mathcal{F}$: if $C_{new}[i]$ is not dominated by any other solution currently contained in $\mathcal{F}$, it is added to $\mathcal{F}$ and all schedules dominated by $C_{new}[i]$ are removed from $\mathcal{F}$. If the ideotype is not yet obtained, C_{new} is added to the queue $\mathcal{Q}$ for further extension, where some alternatives $C_{new}[i]$ may be discarded by one of the applied heuristics or if it is predicted that all extensions will violate the constraints and/or will be dominated by an already obtained solution (pruning). Note that in the actual implementation, some (heuristic) pruning criteria are checked at other points through the execution to enable early pruning (e.g. before combining two specific partial schedules or before attaching a specific genotype G as the next target).

Gene Stacker continues until $\mathcal{Q}$ is empty; termination is guaranteed because of a required constraint on the number of generations. Note that the algorithm is not entirely exact as it greedily discards non Pareto optimal alignments of partial crossing schedules. However, the impact of this greedy approach on the solution quality is expected to be very small; it mainly prevents the introduction of most likely redundant generations in C_{new} when generations of the combined schedules can easily be aligned without violating any constraints, and favors those alignments with the highest amount of reuse, resulting in the lowest total cost.

Table S1: Heuristic presets combining well-chosen heuristics.

Preset	Enabled heuristics	Dual run
Best	none	
Better	H0, H1a, H2a, H3s1	✓
Default	H0, H1a, H2a, H3s1, H4, H5, H6	✓
Faster	H0, H1b, H2b, H3s2, H4, H5c, H6	✓
Fastest	H0, H1b, H2b, H3, H4, H5c, H6	

5 Heuristic presets

Gene Stacker provides five presets that each combine a set of well-chosen heuristics, offering convenient tradeoffs between solution quality and execution time. The specific heuristics included in each preset are listed in Table S1. In the *default* setting some less restrictive heuristics are applied compared to those enabled when switching to presets *faster* and *fastest*. On the other hand, preset *better* drops some heuristics and preset *best* does not apply any (optional) heuristics at all. Presets *better*, *default* and *faster* perform two runs as they apply one of the dual run heuristics H3s1 or H3s2, while preset *fastest* applies H3 in a single run.

6 Results for the first constructed example

Figure S4 shows an overview of all reported solutions for the first constructed example, when running Gene Stacker in *default* mode with an overall success rate of $\gamma = 0.95$ and a maximum of 4 generations, 10% overall linkage phase ambiguity, 4 crossings per plant, 5000 plants per generation, and 2500 seeds obtained from each crossing. Three schedules are non-ambiguous, while the remaining two schedules have a small linkage phase ambiguity of 8.28% which in turn yields a (slightly) lower total population size. The approximated Pareto frontier clearly reflects the tradeoffs between the three objectives: minimizing the total population size, number of generations and overall linkage phase ambiguity.

7 Specification of real stacking problems

This section gives a full description of all discussed problems from cotton, tomato and rice.

7.1 Cotton

One example from cotton is considered. The problem consists of 6 parental genotypes and a heterozygous ideotype with 11 loci spread across 5 chromosomes:

$$G^1 = \begin{bmatrix} 0 \\ 1 \end{bmatrix} \begin{bmatrix} 0 & 0 & 1 \\ 0 & 0 & 1 \end{bmatrix} \begin{bmatrix} 0 & 0 \\ 0 & 0 \end{bmatrix} \begin{bmatrix} 0 & 0 \\ 0 & 0 \end{bmatrix} \begin{bmatrix} 1 & 0 & 0 \\ 1 & 0 & 0 \end{bmatrix},$$

$$G^2 = \begin{bmatrix} 0 \\ 0 \end{bmatrix} \begin{bmatrix} 1 & 1 & 0 \\ 0 & 0 & 0 \end{bmatrix} \begin{bmatrix} 0 & 0 \\ 0 & 0 \end{bmatrix} \begin{bmatrix} 0 & 0 \\ 0 & 0 \end{bmatrix} \begin{bmatrix} 0 & 0 & 0 \\ 0 & 0 & 0 \end{bmatrix},$$

$$G^3 = \begin{bmatrix} 0 \\ 0 \end{bmatrix} \begin{bmatrix} 0 & 0 & 0 \\ 0 & 0 & 0 \end{bmatrix} \begin{bmatrix} 1 & 1 \\ 1 & 1 \end{bmatrix} \begin{bmatrix} 0 & 0 \\ 0 & 0 \end{bmatrix} \begin{bmatrix} 0 & 0 & 0 \\ 0 & 0 & 0 \end{bmatrix},$$

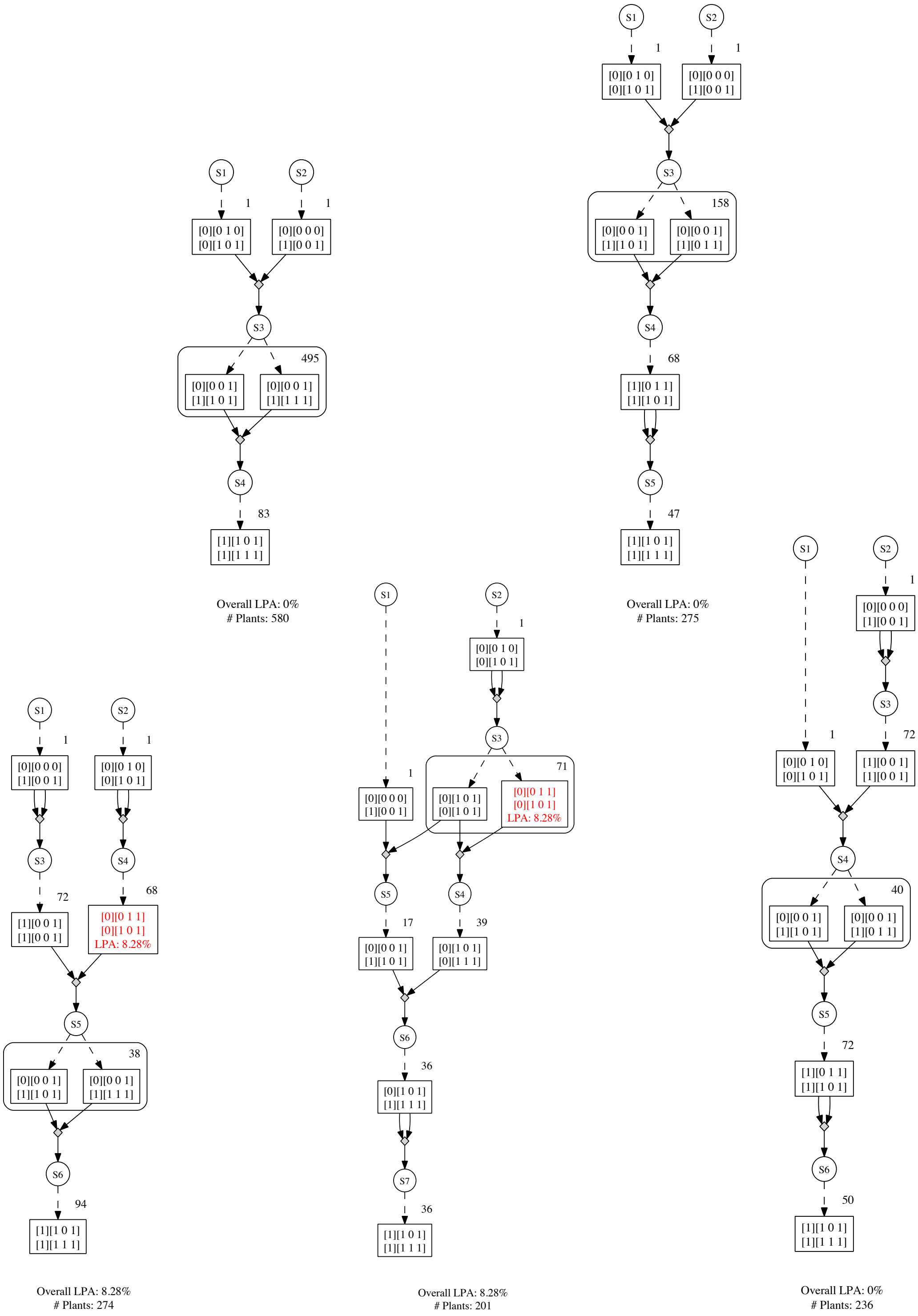

Figure S4: Overview of all reported solutions for the first constructed example, when running Gene Stacker in *default* mode with an overall success rate of $\gamma = 0.95$ and a maximum of 4 generations, 10% overall linkage phase ambiguity, 4 crossings per plant, 5000 plants per generation, and 2500 seeds obtained from each crossing.

$$G^4 = \begin{bmatrix} 0 \\ 0 \end{bmatrix} \begin{bmatrix} 1 & 1 & 0 \\ 0 & 0 & 0 \end{bmatrix} \begin{bmatrix} 1 & 1 \\ 0 & 0 \end{bmatrix} \begin{bmatrix} 0 & 0 \\ 0 & 0 \end{bmatrix} \begin{bmatrix} 0 & 0 & 0 \\ 0 & 0 & 0 \end{bmatrix},$$

$$G^5 = \begin{bmatrix} 0 \\ 0 \end{bmatrix} \begin{bmatrix} 0 & 0 & 0 \\ 0 & 0 & 0 \end{bmatrix} \begin{bmatrix} 0 & 0 \\ 0 & 0 \end{bmatrix} \begin{bmatrix} 1 & 1 \\ 1 & 1 \end{bmatrix} \begin{bmatrix} 0 & 1 & 1 \\ 0 & 1 & 1 \end{bmatrix},$$

$$G^6 = \begin{bmatrix} 0 \\ 0 \end{bmatrix} \begin{bmatrix} 1 & 1 & 0 \\ 1 & 1 & 0 \end{bmatrix} \begin{bmatrix} 0 & 0 \\ 0 & 0 \end{bmatrix} \begin{bmatrix} 1 & 1 \\ 0 & 0 \end{bmatrix} \begin{bmatrix} 0 & 0 & 0 \\ 0 & 0 & 0 \end{bmatrix},$$

$$\mathcal{I} = \begin{bmatrix} 0 \\ 1 \end{bmatrix} \begin{bmatrix} 1 & 1 & 1 \\ 1 & 1 & 1 \end{bmatrix} \begin{bmatrix} 0 & 0 \\ 1 & 1 \end{bmatrix} \begin{bmatrix} 1 & 1 \\ 1 & 1 \end{bmatrix} \begin{bmatrix} 1 & 0 & 0 \\ 1 & 1 & 1 \end{bmatrix}.$$

The genetic map states the following distances between subsequent loci on the same chromosome:

- 2nd chromosome: 15 cM, 10 cM
- 3rd chromosome: 10 cM
- 4th chromosome: 8 cM
- 5th chromosome: 45 cM, 10 cM

7.2 Tomato

Both considered stacking problems from tomato consist of the same 4 parental genotypes with 8 loci spread across 6 chromosomes:

$$G^1 = \begin{bmatrix} 1 \\ 0 \end{bmatrix} \begin{bmatrix} 0 & 0 \\ 0 & 0 \end{bmatrix} \begin{bmatrix} 0 \\ 0 \end{bmatrix} \begin{bmatrix} 0 \\ 0 \end{bmatrix} \begin{bmatrix} 0 \\ 0 \end{bmatrix} \begin{bmatrix} 0 & 0 \\ 0 & 0 \end{bmatrix},$$

$$G^2 = \begin{bmatrix} 0 \\ 0 \end{bmatrix} \begin{bmatrix} 0 & 0 \\ 0 & 0 \end{bmatrix} \begin{bmatrix} 0 \\ 1 \end{bmatrix} \begin{bmatrix} 0 \\ 1 \end{bmatrix} \begin{bmatrix} 0 \\ 1 \end{bmatrix} \begin{bmatrix} 1 & 1 \\ 1 & 1 \end{bmatrix},$$

$$G^3 = \begin{bmatrix} 0 \\ 0 \end{bmatrix} \begin{bmatrix} 0 & 1 \\ 0 & 1 \end{bmatrix} \begin{bmatrix} 0 \\ 0 \end{bmatrix} \begin{bmatrix} 0 \\ 0 \end{bmatrix} \begin{bmatrix} 0 \\ 0 \end{bmatrix} \begin{bmatrix} 0 & 0 \\ 0 & 0 \end{bmatrix},$$

$$G^4 = \begin{bmatrix} 0 \\ 0 \end{bmatrix} \begin{bmatrix} 1 & 0 \\ 1 & 0 \end{bmatrix} \begin{bmatrix} 0 \\ 0 \end{bmatrix} \begin{bmatrix} 0 \\ 0 \end{bmatrix} \begin{bmatrix} 0 \\ 0 \end{bmatrix} \begin{bmatrix} 0 & 0 \\ 0 & 0 \end{bmatrix}.$$

The genetic map states the following distances between subsequent loci on the same chromosome:

- 2nd chromosome: 4 cM
- 6th chromosome: 10cM

The first problem (Tomato-1) has a homozygous ideotype

$$\mathcal{I} = \begin{bmatrix} 1 \\ 1 \end{bmatrix} \begin{bmatrix} 1 & 1 \\ 1 & 1 \end{bmatrix} \begin{bmatrix} 1 \\ 1 \end{bmatrix} \begin{bmatrix} 1 \\ 1 \end{bmatrix} \begin{bmatrix} 1 \\ 1 \end{bmatrix} \begin{bmatrix} 1 & 1 \\ 1 & 1 \end{bmatrix},$$

while the second problem (Tomato-2) has a heterozygous ideotype

$$\mathcal{I} = \begin{bmatrix} 0 \\ 1 \end{bmatrix} \begin{bmatrix} 1 & 1 \\ 1 & 1 \end{bmatrix} \begin{bmatrix} 0 \\ 1 \end{bmatrix} \begin{bmatrix} 0 \\ 1 \end{bmatrix} \begin{bmatrix} 0 \\ 1 \end{bmatrix} \begin{bmatrix} 1 & 1 \\ 1 & 1 \end{bmatrix}.$$

7.3 Rice

The two considered problems from rice have the same 8 parental genotypes with 10 loci spread across 6 chromosomes:

$$
\begin{aligned}
G^1 &= \begin{bmatrix} 0 \\ 0 \end{bmatrix} \begin{bmatrix} 1 \\ 1 \end{bmatrix} \begin{bmatrix} 1 & 1 \\ 1 & 1 \end{bmatrix} \begin{bmatrix} 0 & 0 \\ 0 & 0 \end{bmatrix} \begin{bmatrix} 1 & 1 & 1 \\ 0 & 0 & 0 \end{bmatrix} \begin{bmatrix} 1 \\ 1 \end{bmatrix}, \\
G^2 &= \begin{bmatrix} 1 \\ 0 \end{bmatrix} \begin{bmatrix} 1 \\ 0 \end{bmatrix} \begin{bmatrix} 1 & 1 \\ 0 & 0 \end{bmatrix} \begin{bmatrix} 0 & 0 \\ 0 & 0 \end{bmatrix} \begin{bmatrix} 1 & 0 & 0 \\ 0 & 0 & 0 \end{bmatrix} \begin{bmatrix} 0 \\ 0 \end{bmatrix}, \\
G^3 &= \begin{bmatrix} 0 \\ 0 \end{bmatrix} \begin{bmatrix} 1 \\ 1 \end{bmatrix} \begin{bmatrix} 1 & 1 \\ 1 & 1 \end{bmatrix} \begin{bmatrix} 0 & 0 \\ 0 & 0 \end{bmatrix} \begin{bmatrix} 1 & 0 & 0 \\ 1 & 0 & 0 \end{bmatrix} \begin{bmatrix} 0 \\ 0 \end{bmatrix}, \\
G^4 &= \begin{bmatrix} 0 \\ 0 \end{bmatrix} \begin{bmatrix} 1 \\ 1 \end{bmatrix} \begin{bmatrix} 1 & 1 \\ 1 & 1 \end{bmatrix} \begin{bmatrix} 0 & 0 \\ 0 & 0 \end{bmatrix} \begin{bmatrix} 0 & 0 & 0 \\ 0 & 0 & 0 \end{bmatrix} \begin{bmatrix} 1 \\ 1 \end{bmatrix}, \\
G^5 &= \begin{bmatrix} 1 \\ 0 \end{bmatrix} \begin{bmatrix} 1 \\ 0 \end{bmatrix} \begin{bmatrix} 1 & 1 \\ 0 & 0 \end{bmatrix} \begin{bmatrix} 0 & 0 \\ 0 & 0 \end{bmatrix} \begin{bmatrix} 0 & 0 & 0 \\ 0 & 0 & 0 \end{bmatrix} \begin{bmatrix} 0 \\ 0 \end{bmatrix}, \\
G^6 &= \begin{bmatrix} 0 \\ 0 \end{bmatrix} \begin{bmatrix} 1 \\ 1 \end{bmatrix} \begin{bmatrix} 1 & 1 \\ 1 & 1 \end{bmatrix} \begin{bmatrix} 0 & 0 \\ 0 & 0 \end{bmatrix} \begin{bmatrix} 0 & 0 & 0 \\ 0 & 0 & 0 \end{bmatrix} \begin{bmatrix} 0 \\ 0 \end{bmatrix}, \\
G^7 &= \begin{bmatrix} 0 \\ 0 \end{bmatrix} \begin{bmatrix} 1 \\ 1 \end{bmatrix} \begin{bmatrix} 1 & 1 \\ 1 & 1 \end{bmatrix} \begin{bmatrix} 1 & 1 \\ 1 & 1 \end{bmatrix} \begin{bmatrix} 1 & 0 & 0 \\ 1 & 0 & 0 \end{bmatrix} \begin{bmatrix} 0 \\ 0 \end{bmatrix}, \\
G^8 &= \begin{bmatrix} 1 \\ 1 \end{bmatrix} \begin{bmatrix} 0 \\ 0 \end{bmatrix} \begin{bmatrix} 0 & 0 \\ 0 & 0 \end{bmatrix} \begin{bmatrix} 0 & 0 \\ 0 & 0 \end{bmatrix} \begin{bmatrix} 0 & 0 & 0 \\ 0 & 0 & 0 \end{bmatrix} \begin{bmatrix} 0 \\ 0 \end{bmatrix}.
\end{aligned}
$$

The genetic map states the following distances between subsequent loci on the same chromosome:

- 3rd chromosome: 5 cM
- 4th chromosome: 9 cM
- 5th chromosome: 50 cM, 8 cM

The first problem (Rice-1) has a homozygous ideotype

$$
\mathcal{I} = \begin{bmatrix} 1 \\ 1 \end{bmatrix} \begin{bmatrix} 1 \\ 1 \end{bmatrix} \begin{bmatrix} 1 & 1 \\ 1 & 1 \end{bmatrix} \begin{bmatrix} 1 & 1 \\ 1 & 1 \end{bmatrix} \begin{bmatrix} 1 & 1 & 1 \\ 1 & 1 & 1 \end{bmatrix} \begin{bmatrix} 1 \\ 1 \end{bmatrix},
$$

while the second problem (Rice-2) has a heterozygous ideotype

$$
\mathcal{I} = \begin{bmatrix} 1 \\ 1 \end{bmatrix} \begin{bmatrix} 1 \\ 1 \end{bmatrix} \begin{bmatrix} 1 & 1 \\ 1 & 1 \end{bmatrix} \begin{bmatrix} 0 & 1 \\ 1 & 1 \end{bmatrix} \begin{bmatrix} 0 & 0 & 0 \\ 1 & 1 & 1 \end{bmatrix} \begin{bmatrix} 1 \\ 1 \end{bmatrix}.
$$

8 Solutions for real stacking problem from cotton

Figures S5 up to S8 show all solutions reported for the cotton example when applying preset *fastest* with a maximum of 5 generations. Running this preset took 2 hours and 15 minutes to complete; all other presets ran out of memory.

As the number of seeds obtained from a crossing is set to 250 some crossings are performed multiple times to provide a sufficient amount of seeds so that all targeted genotypes can be obtained among the offspring. Furthermore, a cotton plant can only be crossed twice (or selfed once); therefore, for some genotypes several duplicates are targeted to be able to perform all crossings. Population

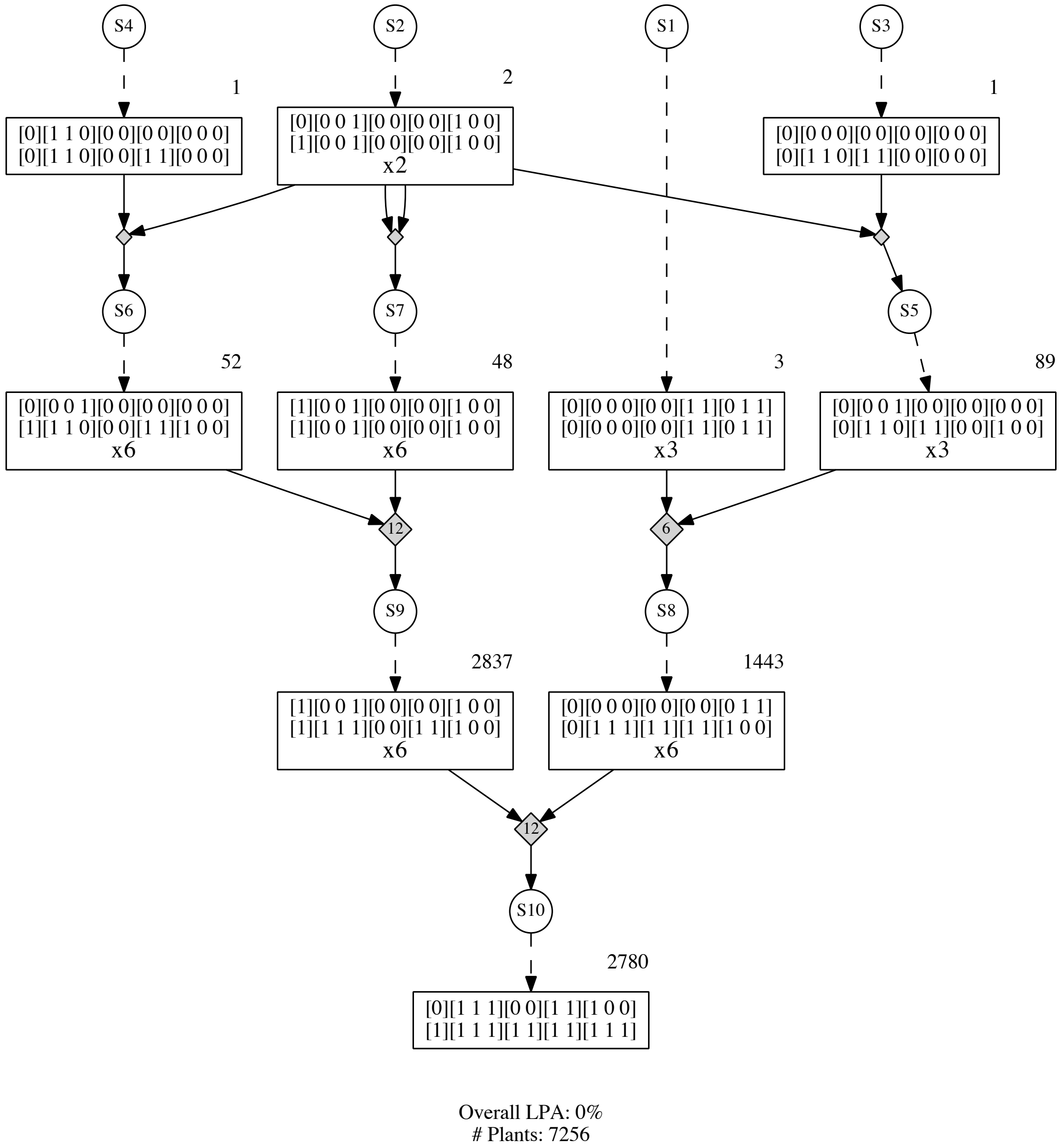

Figure S5: Three generation solution for the cotton example. Some crossings are performed 6 up to 12 times to provide a sufficient amount of seeds. For most genotypes occurring in the schedule, several duplicates are targeted to be able to perform all crossings.

sizes are computed in such way that at least the required number of instances is expected among the offspring, for each targeted genotype (see section 2).

When restricting the number of generations to 4 instead of 5, preset *faster* reports a different solution with four generations (Figure S9) that has a lower total population size compared to the respective schedule found by preset *fastest*, before being interrupted when the time limit of 24 hours is exceeded.

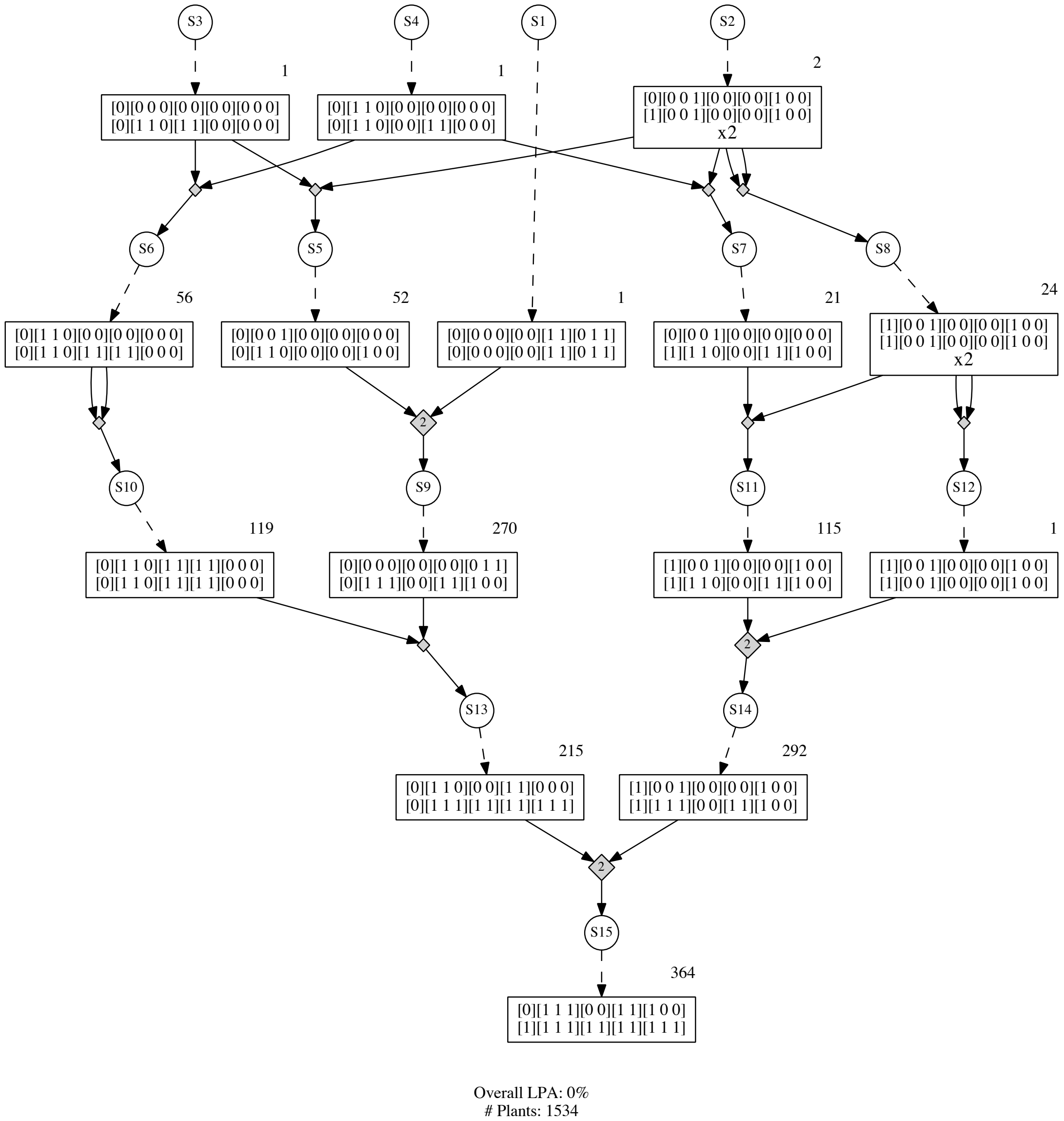

Figure S6: Four generation solution for the cotton example. Some crossings are performed twice to obtain a sufficient amount of seeds. For two genotypes occurring in the schedule, two duplicates are targeted to be able to perform all crossings.

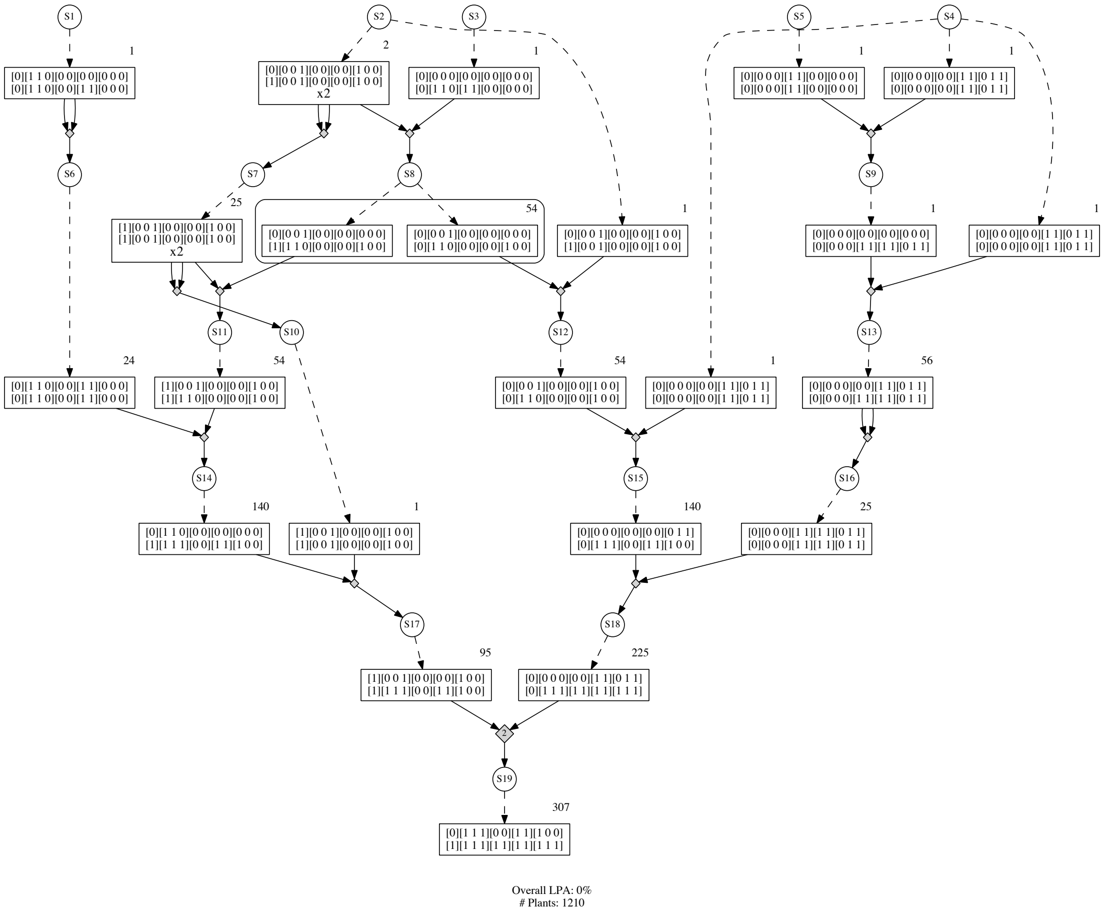

Figure S7: Five generation solution for the cotton example with zero linkage phase ambiguity. Only the final crossing is performed twice to provide a sufficient amount of seeds. For two genotypes occurring in the schedule, two duplicates are targeted to be able to perform all crossings.

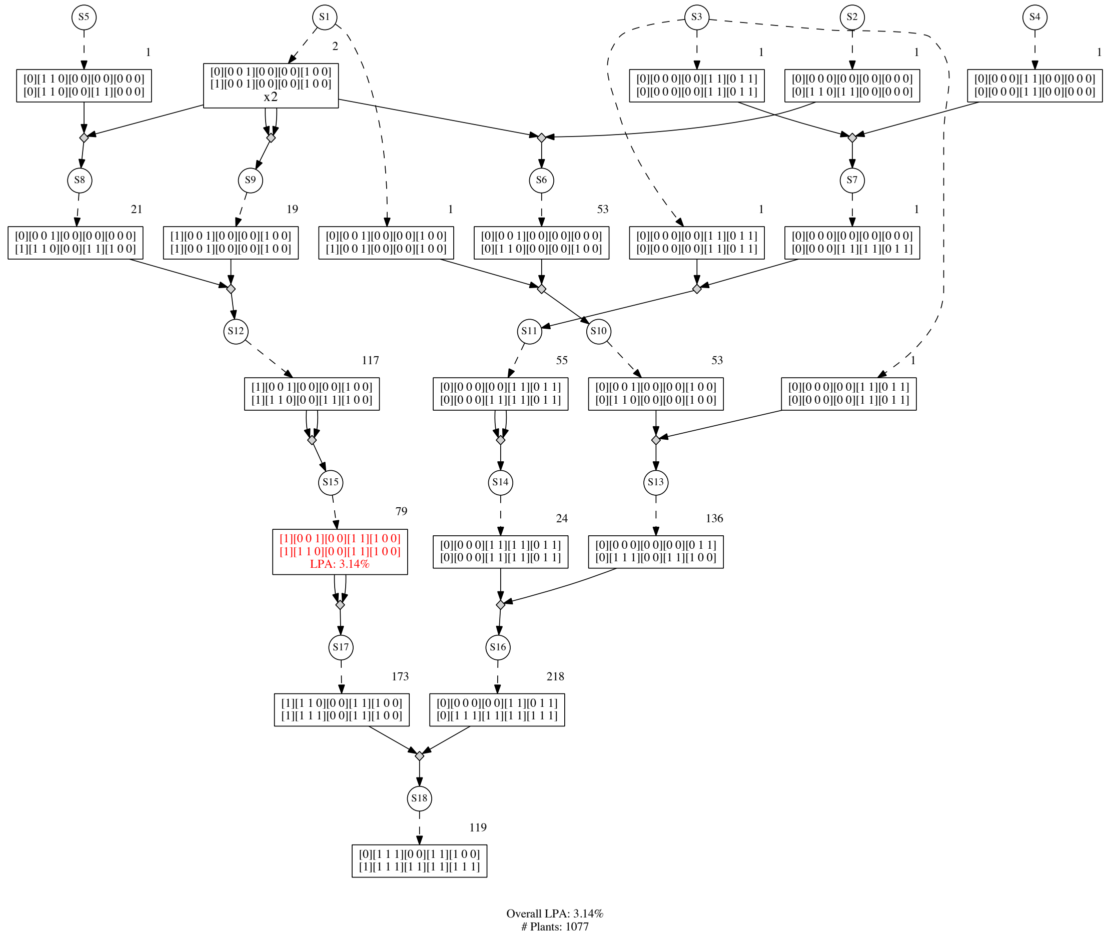

Figure S8: Five generation solution for the cotton example with an overall linkage phase ambiguity of 3.14%. In return, a reduction in the total population size is obtained compared to the reported non-ambiguous schedule with five generations. No crossings are performed multiple times in this schedule as a single crossing always provides enough seeds to obtain all targeted genotypes.

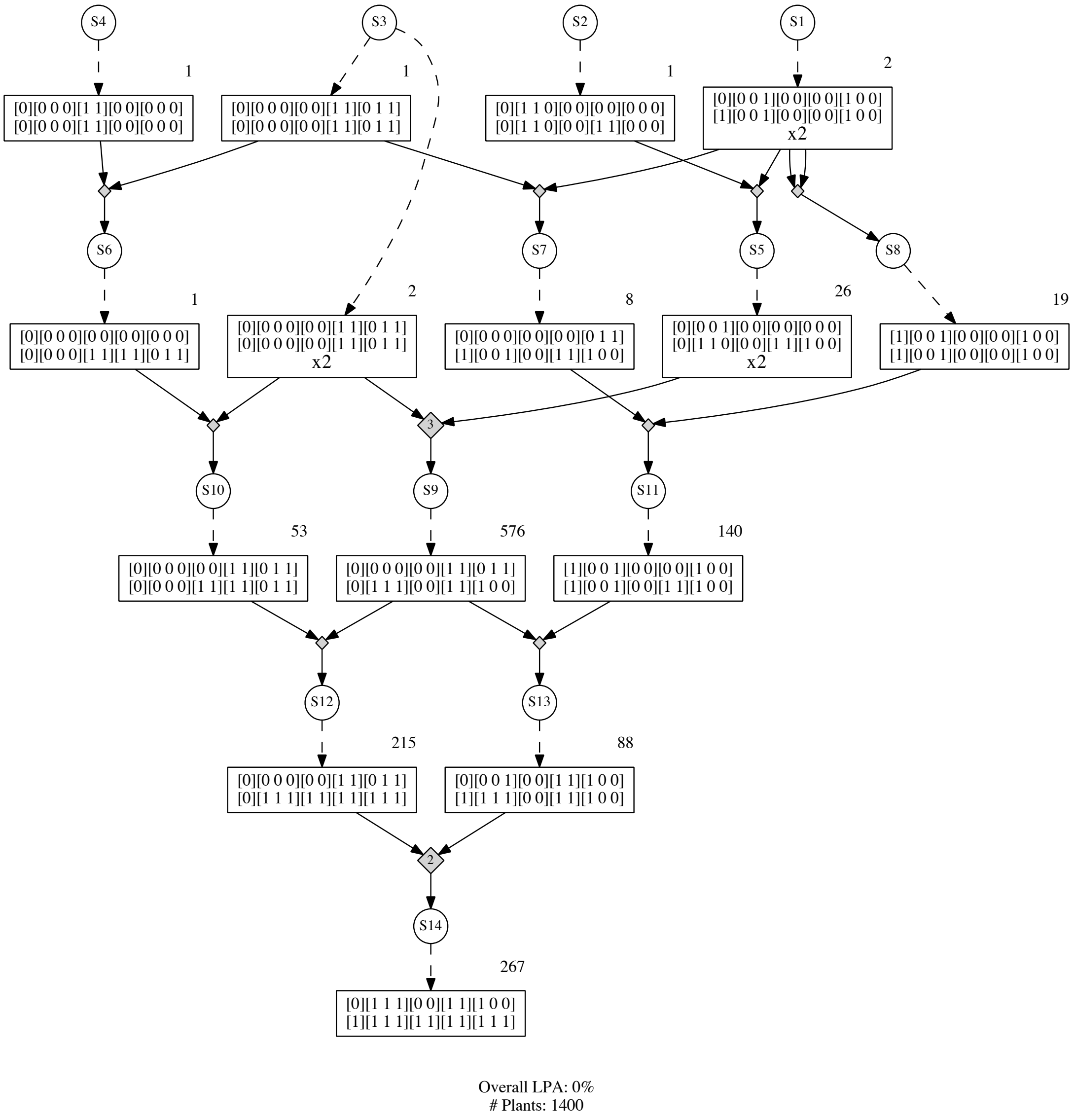

Figure S9: Additional four generation solution for the cotton example, reported by preset *faster* when restricting the number of generations to 4 instead of 5. This schedule has a lower total population size compared to the solution with four generations that is found by preset *fastest*.

References

[1] Stefan Canzar and Mohammed El-Kebir. A mathematical programming approach to marker-assisted gene pyramiding. In Teresa M Przytycka and Marie-France Sagot, editors, *Algorithms in Bioinformatics, WABI 2011, LNBI 6833*, pages 26–38, Heidelberger Platz 3, 14197 Berlin, Germany, 2011. Springer.
